# Design and Synthesis of Newly Synthesized Acrylamide Derivatives as Potential Chemotherapeutic Agents against MCF-7 Breast Cancer Cell Line Lodged on PEGylated Bilosomal Nano-Vesicles for Improving Cytotoxic Activity

**DOI:** 10.3390/ph14101021

**Published:** 2021-10-04

**Authors:** Islam Zaki, Reham A. I. Abou-Elkhair, Ali H. Abu Almaaty, Ola A. Abu Ali, Eman Fayad, Ahmed Gaafar Ahmed Gaafar, Mohamed Y. Zakaria

**Affiliations:** 1 Pharmaceutical Organic Chemistry Department, Faculty of Pharmacy, Port Said University, Port Said 42526, Egypt; Eslam.zaki@pharm.psu.edu.eg; 2 Applied Nucleic Acids Research Center & Chemistry Department, Faculty of Science, Zagazig University, Zagazig 44523, Egypt; abdallaelsayed@zu.edu.eg; 3 Zoology Department, Faculty of Science, Port Said University, Port Said 42526, Egypt; ali_zoology_2010@yahoo.com; 4 Chemistry Department, College of Science, Taif University, P.O. Box 11099, Taif 21944, Saudi Arabia; O.abuali@tu.edu.sa; 5 Biotechnology Department, Faculty of Sciences, Taif University, P.O. Box 11099, Taif 21944, Saudi Arabia; e.esmail@tu.edu.sa; 6 Pharmacology and Toxicology Department, Faculty of Pharmacy, Port Said University, Port Said 42526, Egypt; ahmed.gafar@pharm.psu.edu.eg; 7 Department of Pharmaceutics and Industrial Pharmacy, Faculty of Pharmacy, Port Said University, Port Said 42526, Egypt

**Keywords:** acrylamide, tubulin, cell cycle analysis, annexin, PEGylated bilosomes, aqueous solubility and optimization

## Abstract

Cancer is a multifaceted disease. With the development of multi drug resistance, the need for the arousal of novel targets in order to avoid these drawbacks increased. A new series of acrylamide derivatives was synthesized from starting material 4-(furan-2-ylmethylene)-2-(3,4,5-trimethoxyphenyl)oxazol-5(4*H*)–one (1), and they are evaluated for their inhibitory activity against β-tubulin polymerization. The target molecules 2–5 d were screened for their cytotoxic activity against breast cancer MCF-7 cell line. The results of cytotoxicity screening revealed that compounds 4e and 5d showed good cytotoxic profile against MCF-7 cells. Compounds 4e produced significant reduction in cellular tubulin with excellent β-tubulin polymerization inhibition activity. In addition, compound 4e exhibited cytotoxic activity against MCF-7 cells by cell cycle arrest at pre-G1 and G2/M phases, as shown by DNA flow cytometry assay. Aiming to enhance the limited aqueous solubility and, hence, poor oral bioavailability of the prepared lead acrylamide molecule, 4e-charged PEGylated bilosomes were successfully fabricated via thin film hydration techniques as an attempt to improve these pitfalls. 2^3^ full factorial designs were manipulated to examine the influence of formulation variables: types of bile salt including either sodium deoxy cholate (SDC) or sodium tauro cholate (STC), amount of bile salt (15 mg or 30 mg) and amount of DSPE–mPEG-2000 amount (25 mg or 50 mg) on the characteristics of the nanosystem. The F7 formula of entrapment efficiency (E.E% = 100 ± 5.6%), particle size (PS = 280.3 ± 15.4 nm) and zeta potential (ZP = −22.5 ± 3.4 mv) was picked as an optimum formula with a desirability value of 0.868. Moreover, prominent enhancement was observed at the compound’s cytotoxic activity (IC_50_ = 0.75 ± 0.03 µM) instead of (IC_50_ = 2.11 ± 0.19 µM) for the unformulated 4e after being included in the nano-PEGylated bilosomal system.

## 1. Introduction

Microtubules are one of the most important cellular protein scaffolds [1,2]. Microtubules along with actin and intermediate filaments are major cell building blocks and, therefore, play an integral role in cell reproductive processes during mitosis [3,4]. Microtubules are also crucial for a variety of fundamental cell processes, including cell proliferation, sustained cell shape and structure, intracellular transport of vesicles and protein complexes and motility regulation [5,6,7]. Additionally, the disruption of microtubules can induce cell cycle arrest in the G2/M phase, formation of abnormal mitotic spindles and final triggering of signals for apoptosis [8,9,10]. Therefore, the importance of microtubules in mitosis and cell division makes them an attractive target for the development of anticancer drugs.

Breast cancer characteristically displays uncontrolled or abnormal cell proliferation due to excessive microtubule synthesis [11,12]. Knowledge and understanding of this intrinsic property have resulted in the development of chemotherapeutic regimens that act by interfering with the microtubule assembly or disassembly [13].

**Figure 1:**
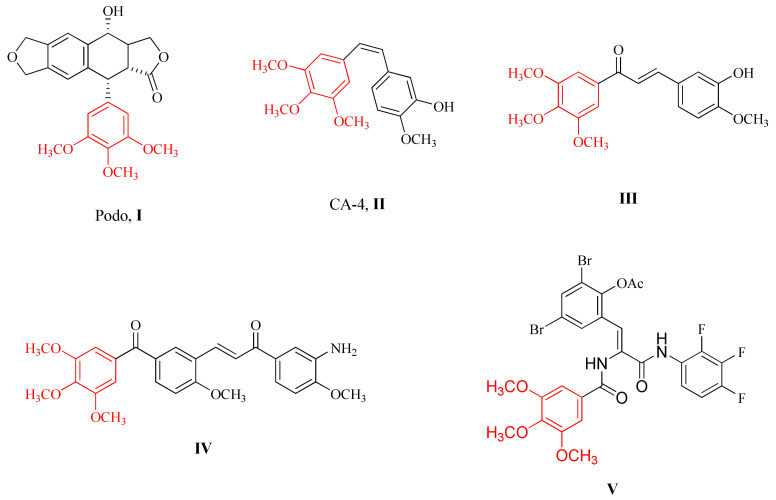
Chemical structures of antimitotic agents **I**–**V**.

Antimitotic agents such as podophyllotoxin (podo) **I**, combretastatin A-4 (CA-4) **II** and chalcone **III** (Figure 1) exhibited good cytotoxicity profile due to strong tubulin polymerization inhibition activity [14,15,16,17]. Compound **IV** displayed a broad spectrum of antiproliferative activity on most of the cell lines of NCI in the sub-micromolar range and exhibited significant inhibitory effect on the tubulin assembly with an IC_50_ value of 0.6 μM [18]. In addition, compound **V** showed potent inhibition of tubulin polymerization and arrested the cell at the G2/M phase of the cell cycle compared with reference compound CA-4 [19].

Unfortunately, most of chemotherapeutic drugs that suffer from a lack of persistent clinical and therapeutic outcomes. Moreover, they are associated with extensive adverse effects and diminished bioavailability [20]. In order to eliminate these obstacles, the emergence of novel drug delivery systems based on nanotechnology such as liposomes, polymeric nanoparticles and micelles, etc., becomes essential [20,21]. However, conventional vesicular systems such as liposomes suffer from diminished encapsulation capability, stability, encapsulation and vast problems associated with scaling up problems, which provoke the necessity for the evolution of de novo vesicular systems [22,23].

The incorporation of bile salt in the vesicular structure aims to bypass the stability issue and other drawbacks associated with the other conventional vesicular systems, particularly for liposomes and niosomes [24]. Bilosomes have been manipulated for orally dispensed drugs possessing faint water solubility and reduced stability versus harsh conditions in GIT [24]. Moreover, PEGylated vesicles propose more advantages over nude vesicles such as restrained drug release manner, extended drug circulation time in systemic circulation and being as a shelter that suppresses the possibility of vesicles adhesion with plasma proteins [21].

**Figure 2:**
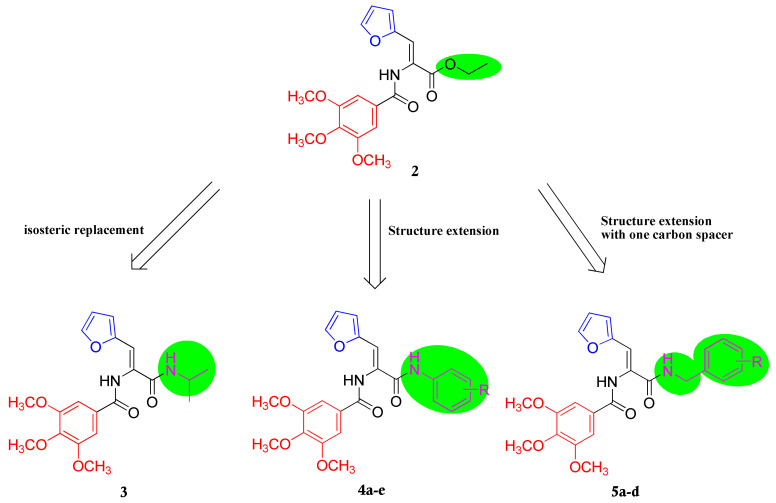
Design of the target anticancer acrylamide derivatives **3**–**5d**.

Based on the foregoing aspects and in continuation of the efforts to discover anticancer agents [25,26,27,28,29], a mimic anticancer model was designed based on a diamide scaffold. The model has the following structural outline: triaryl rings connected through two amide groups. One of the aryl ring attached to the amide group composed of 3,4,5-trimethoxy phenyl (TMP) moiety in order to mimic TMP ring in compounds **I**–**V**. The incorporation of furan moiety as the second aryl ring into the diamide scaffold was performed to augment the anticancer activity of the produced compounds since oxygen in furan ring has the potential for hydrogen bonding with the target [30]. Finally, the third aryl ring contains varying substituents of aliphatic, aryl substituted or substituted benzyl rings for comparative reasons to investigate the impact of these modifications on the activity. The difference in the structural variations between the prepared molecules is outlined as follows: starting with ester derivative **2**, the ester moiety could be replaced with *N*-isopropyl amide group to provide compound **3**. Furthermore, the ester group could be replaced with substituted phenyl rings either directly to obtain compounds **4a**–**e** or through one carbon spacer to provide compounds **5a**–**d**. Figure 2 illustrates the design strategy for the structural modification of the target molecules. Furthermore, the inclusion of the most potential lead within an optimized PEGylated bilosomal formulation acquired promising results regarding promoted cytotoxic activity, drug solubility, release and, to an extent, its pharmaceutical properties, which mainly includes the drug’s aqueous solubility and bioavailability.

## 2. Results and Discussion

### 2.1. Chemistry

**Scheme 1:**
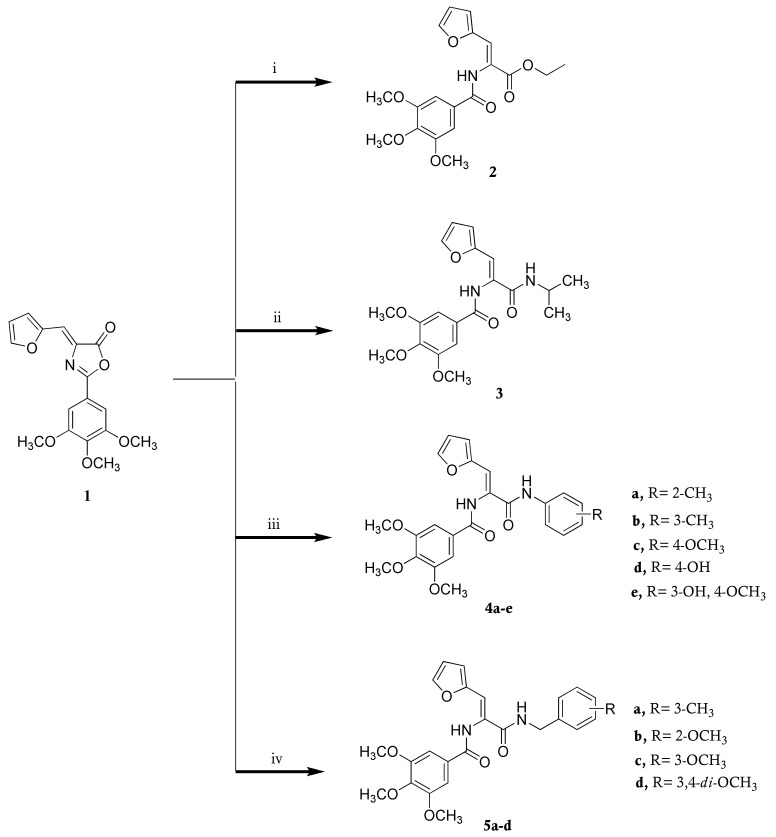
Synthesis of the target compounds **2**–**5d.** Reagents and conditions: **i.** EtOH, Et_3_N, 2 h; **ii.** *iso-*propyl amine, EtOH, 4 h; **iii.** Aromatic amines, EtOH, 4 h; **iv.** Benzyl amines, NaOAc, AcOH, 1 h.

The designed molecules **2**–**5d** were obtained as outlined in Scheme 1. 4-(furan-2-ylmethylene)-2-(3,4,5-trimethoxyphenyl)oxazol-5(4*H*)-one (**1**) is the starting material and was prepared by condensation of 2-(3,4,5-trimethoxybenzamide)acetic acid, furfural and acetic anhydride in the presence of anhydrous sodium acetate in an oil bath at 80–100 °C according to literature reported [25]. The structure of the starting oxazolone **1** was confirmed by IR, ^1^H-NMR and ^13^C-NMR spectra. IR spectrum of oxazolone **1** showed the characteristic C=O stretching absorption of lactone ring at ν 1805 cm^−1^. The ester derivative **2** was prepared by reaction of oxazolone **1** in refluxing absolute ethanol in the presence of triethylamine (Et_3_N). The structure of ester molecule was ascertained on the basis of IR, ^1^H-NMR and ^13^C-NMR spectra. The ^1^H-NMR spectrum revealed the presence of characteristic triplet and quartet signals related to -OCH_2_CH_3_, in addition to an exchangeable proton (NH) at δ 9.89 ppm. Additionally, the reaction of oxazolone **1** with isopropylamine in absolute ethanol at reflux temperatures furnished *N*-[1-(furan-2-yl)-3-(isopropylamino)-3-oxoprop-1-en-2-yl]-3,4,5-trimethoxybenzamide (**3**). The ^1^H-NMR spectrum of compound **3** revealed the presence of doublet and multiplet signals at δ 1.12 and 3.94–4.04 ppm related to two methyl (2CH_3_) and methylidene (CH) of isopropyl group, respectively, in addition to two exchangeable protons (2NH) at δ7.78 and 9.67 ppm. Furthermore, compounds **4a**–**e** were obtained from the reaction of oxazolone **1** with appropriate primary aromatic amines in absolute ethanol. The structures of compounds **4a**–**e** were confirmed using IR and NMR spectra. ^1^H-NMR spectrum of compound **4c** as a representative example showed the presence of characteristic two exchangeable proton (2NH) at δ 9.94 ppm. The ^13^C-NMR spectrum of compound **4c** showed the presence of extra signals related to aromatic carbons. Finally, oxazolone **1** upon treatment with various substituted benzyl amines in glacial acetic acid in the presence of anhydrous sodium acetate produced the corresponding diamide derivatives **5a**–**d**. The structures of compounds **5a**–**d** were confirmed on the basis of IR, ^1^H-NMR and ^13^C-NMR spectral data. ^1^H-NMR spectra of compounds **5a**–**d** revealed the presence of doublet signal assigned to methylene CH_2_ at δ 4.33–4.37 ppm. Further structural evidence of compounds **5a**–**d** stemmed from the ^13^C-NMR spectra, which showed the presence of peaks at δ 37.74–42.55 ppm related to CH_2_ carbon in addition to other signals due to aromatic carbons.

### 2.2. Biology

#### 2.2.1. Cytotoxic Activity against Breast Cancer Cell Line MCF-7

Compounds **2**–**5d** were evaluated for their cytotoxic activities against breast cancer MCF-7 cell line by using an MTT colorimetric assay. The obtained results expressed as IC_50_ values were summarized in Table 1. Cisplatin was used as reference drug in this study. The obtained results of the tested molecules showed moderate to potent anticancer activity. The ester derivative **2** revealed weak activity (IC_50_ > 50 μM) that is increased in the *N*-isopropylamide derivative **3** (IC_50_ = 4.73 μM). Furthermore, *N*-aryl amide derivatives **4a**–**e** exhibited IC_50_ = 2.11–26.83 μM. Compound **4e** (IC_50_ = 2.11 μM) showed superior activity at the submicromolar level than other derivatives **4a**–**d**. Regarding *N*-benzyl amide derivatives **5a**–**d**, 3,4-dimethoxybenzyl amide derivative **5c** (IC_50_ = 2.61 μM) displayed higher cytotoxic activity against MCF-7 compared to other *N*-benzyl amide derivatives. Additionally, the therapeutic safety of *N*-(3-hydroxy-4-methoxy) aryl amide derivative **4e** was determined by screening its cytotoxic activity against normal breast cell line (MCF-10A) using an MTT colorimetric assay. The results found that compound **4e** revealed mild cytotoxic effects (IC_50_ = 29.27 μM). These results indicate the selectivity of *N*-(3-hydroxy-4-methoxy) aryl amide derivative **4e** for tumor cells and its relative safety for normal breast cells.

#### 2.2.2. Tubulin Polymerization Inhibition Assays

**Figure 3:**
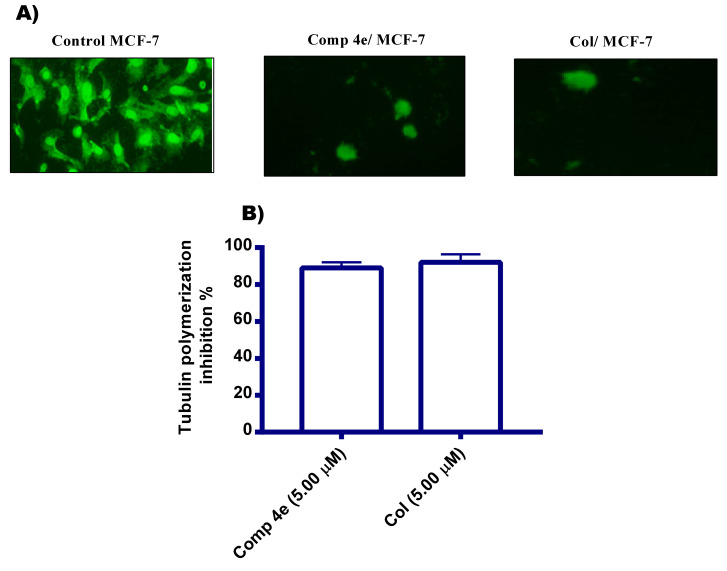
(**A**) Immunofluorescence intensity of tubulin localization in MCF-7 cells after treatment with compound **4e** and Col compared with untreated control cells. (**B**) Tubulin inhibition percentage (%) compared to Col.

In order to explore the cytotoxic activity of the prepared molecules for inhibition of tubulin polymerization, the localization tubulins were visualized in MCF-7 cells after treatment with compound **4e** at its IC_50_ concentration (2.11 μM) and Col at a concentration of 5 μM for 48 h and then submitted to immunofluorescence analysis under apotome fluorescence microscope. As shown in Figure 3A, compound **4e**-treated MCF-7 cells showed abnormal tubulin expression patterns, and the cell–cell microtubules mesh is reduced and fragmented with malformed distribution compared with the untreated control.

In addition, the β-tubulin inhibition percentage was quantized using an ELISA assay after treatment with compounds **4e** and colchicine (Col) using 5 μM concentration. β-tubulin inhibition analysis showed that compound **4e** had good β-tubulin inhibition activity with 89% inhibition percentage that was nearly equipotent to Col, which had β-tubulin inhibition activity ≈ 93% (Figure 3B). The results in this experiment suggested that the mechanism of cytotoxicity of compound **4e** might result from the inhibition of tubulin polymerization.

#### 2.2.3. DNA Flow Cytometry Analysis

##### Cell Cycle Analysis

**Figure 4:**
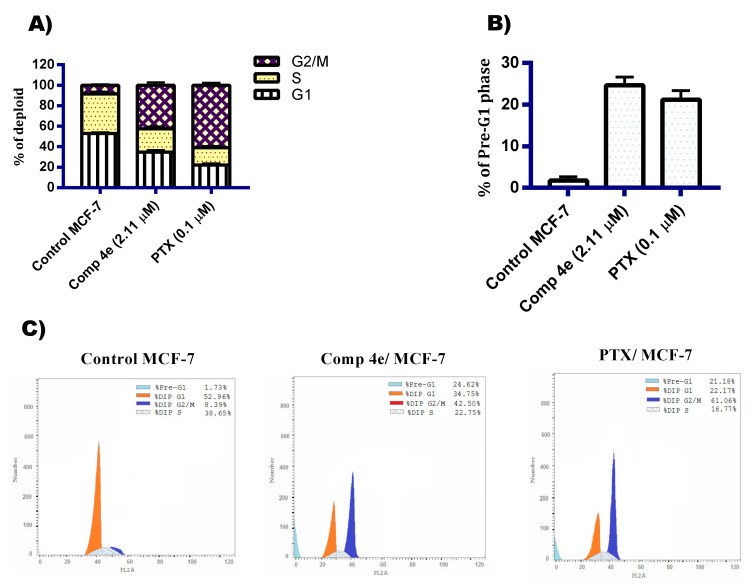
(**A**) Graphical representation of cell cycle analysis of compound **4e** and PTX compared with control cells. (**B**) Graphical representation of the percentage of pre-G1 phase after treatment with compound **4e** and PTX compared with untreated control cells. (**C**) Effect of compound **4e** and PTX on the cell cycle profile compared with untreated control cells.

In order to study the mechanism of the cytotoxic activity of compound **4e,** cell cycle analysis was investigated in breast cancer MCF-7 cells by DNA flow cytometry analysis at IC_50_ dose values of compound **4e** for 48 h. Paclitaxel (PTX, 0.1 μM) was used as the reference drug in this study. As revealed in Figure 4, exposure of MCF-7 cells to compound **4e** resulted in interference of the normal cell distribution in the cell cycle profile of MCF-7 cells. Compound **4e** increased the percentage of cells in G2/M phase from 8.39% to 42.50% compared with the untreated control sample. This effect was accompanied by an increase in the percentage of cells in pre-G_1_ phase from 1.73% to 24.62%. Moreover, the capability of compound **4e** in the induction of apoptosis in MCF-7 cells was superior over that of PTX. These results suggested that compound **4e** induced cancer cell death via G2/M phase arrest with apoptosis inducing activity marked by the presence pre-G_1_ peak in the cell cycle profile of MCF-7 cells, and it is in line with the previously discussed data.

##### Annexin V/ FITC Apoptosis Staining Assay

**Figure 5:**
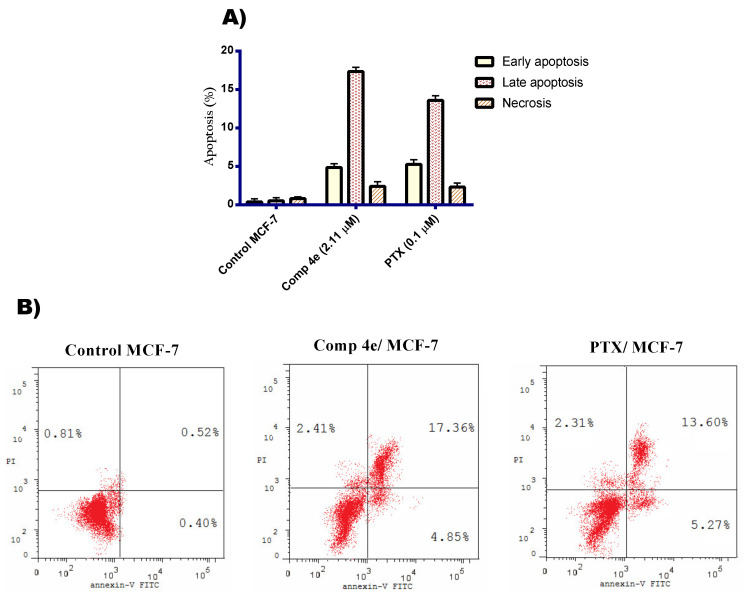
(**A**) Graphical representation of Annexin V/ PI analysis of compound **4e** and PTX compared with untreated control cells. (**B**) Annexin V/PI analysis of compound **4e** and PTX compared with untreated control cells.

Apoptosis is a process of cell death with characteristic morphological and biochemical features distinguishable from those associated with necrosis or accidental death [19]. In order to determine the apoptosis inducing activity of compound **4e,** a biparametric cytofluorimetric analysis was performed for the selected candidate at IC_50_ (2.11 μM) using AnnexinV/Propidium iodide (PI). The obtained data in Figure 5 obviously indicate that compound **4e** increased the percentage of cells at early and late stages compared to the untreated control. The percentage of early apoptotic cells increased by 6.23-fold compared to the untreated control. In addition, compound **4e** increased the percentage of late apoptotic cells by 21.10-fold more than the untreated control. The data in this study indicate that compound **4e** is an effective inducer of apoptosis in MCF-7 cells.

#### 2.2.4. Caspase 3/7 Assay of Compound **4e**

**Figure 6:**
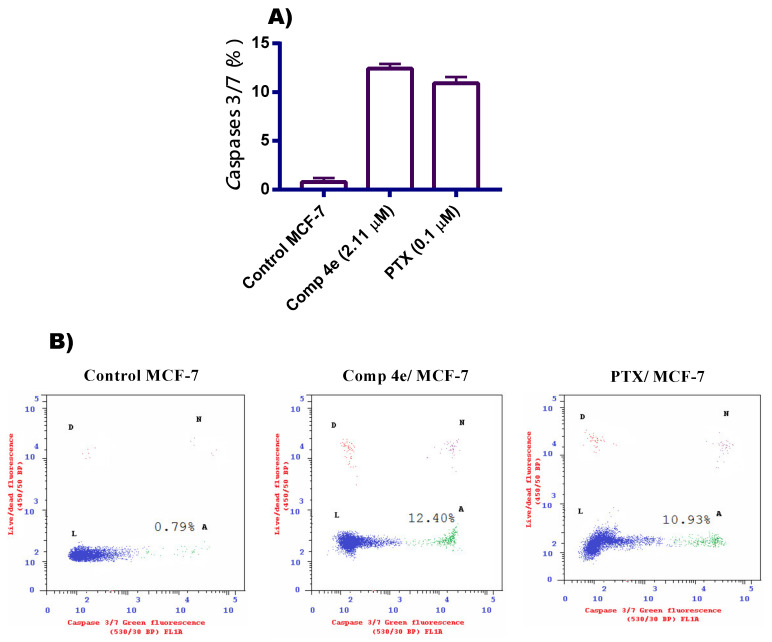
(**A**) Graphical representation of *c*aspase 3/7 analysis related to apoptosis of compound **4e** and PTX compared with untreated control cells. (**B**) *C*aspase 3/7 analysis related to apoptosis of compound **4e** and PTX compared with untreated control cells.

To confirm the apoptosis induced by compound **4e,** *c*aspase 3/7 stimulation assay were carried out for compound **4e** at its IC_50_ (2.11 μM) by using green flow cytometry analysis. The results showed that compound **4e** highly stimulated *c*aspase 3/7 by 6.95-fold more than the control. This experiment confirms that compound **4e** stimulates *c*aspase-3/7 activation and, hence, is involved in apoptosis induction in the MCF-7 cells (Figure 6).

#### 2.2.5. Molecular Docking Study

**Figure 7:**
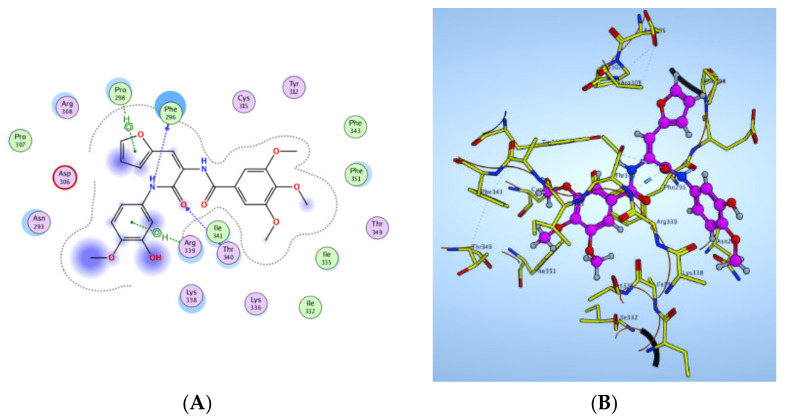
(**A**) Two-dimensional representation of compound **4e** docking into the colchicine binding site. (**B**) Three-dimensional representation of compound **4e** docking into the colchicine binding site.

Molecular docking simulation was performed for compound **4e** into the active site of colchicine binding site in the tubulin protein (PDB code: 1SA0) in order to assess the possible binding interactions with the amino acid residue of the target protein. Figure 7 showed that compound **4e** interacts through hydrogen bonding with the amino acids Thr 340 and Phe 296 by its C=O and amide NH groups, respectively. It also interacts through hydrophobic interaction by its furan and 3-hydroxy-4-methoxyphenyl functions with the amino acid Pro 298 and Arg 339, respectively. Furthermore, the hydrophobicity of *N*-(3-hydroxy-4-methoxy) aryl amide molecule **4e** resulted in the docking score of −13.31 kcal/mol. These findings revealed remarkably high binding affinity of *N*-(3-hydroxy-4-methoxy) aryl amide molecule **4e** into the colchicine binding site of tubulin protein.

### 2.3. Evolvement of PEGylated Bilosomal Nano-Vesicles

#### 2.3.1. In Silico Predictive ADME Study for Targeted Compound (**4e**)

**Figure 8:**
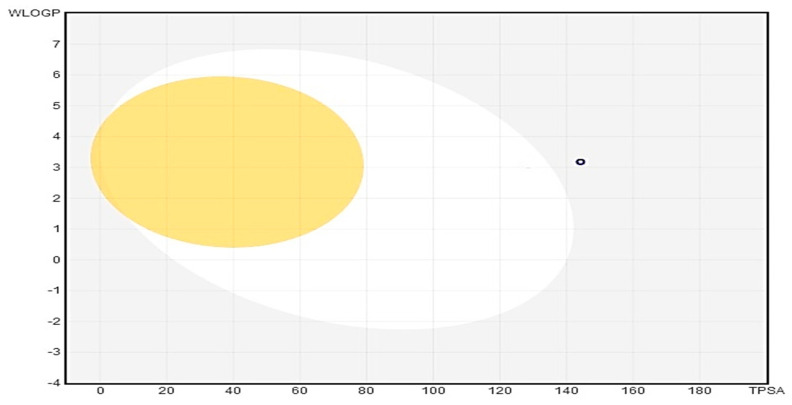
In silico ADME predictive plot: yellow portion indicates blood–brain barrier (BBB) permeability level, while the white portion indicated human intestinal absorption (HIA) level for **4e**.

The exploration of drug-likeness of compound and in silico pharmacokinetic properties was conducted via free web-based tool Swiss ADME (http://www.swissadme.ch/, accessed on 7 July 2021). These studies are correlated to the chemical structure of **4e,** where certain parameters investigated included aqueous solubility level (LogS-SILICOS-IT), absorption Level, atom-based Log P (LogP 98), 2D polar surface area (2D TPSA), blood–brain barrier level (BBB LEV) and the probability of cytochrome P450 2D6 (CYP PROB) and cytochrome P450 2D6 (CYP2D6). The obtained results were depicted as an ADMET plot in Figure 8 by using calculated TPSA-2D and W log P 98 properties.

BBB and human intestinal absorption (HIA) plots were examined for 4e. Figure 8 reveals the BBB plot and **4e** fell outside the 99% and 95% ellipses, as the points on the Figure 8 resembles the molecules predicted to passively permeate through the BBB which indicates that it may not be ready to penetrate the blood–brain barrier. Moreover, the blue circle in Figure 8 denoted that the drug molecule was a substrate relative to P-glycoproteins, and it can be effluxed by it from CNS. Consequently, **4e** may be anticipated to possess low CNS side effects. Consequently, 4e may be anticipated to possess low CNS side effects. Regarding the HIA plot, Figure 8 denotes that **4e** is predicted to have moderate intestinal absorption due to higher number of molecules that can passively penetrate the intestine compared to that of BBB. Stunningly, the predicted poor water solubility of **4e** (LogS-SILICOS-IT = −6.53; 1.39 × 10^−4^ mg/mL; 2.96 × 10^−7^ mol/L) was distinguished as a significant parameter that might impede the compound’s full potential cytotoxic activity and oral bioavailability. This was affirmed by the predicted oral bioavailability (PSA = 145.2). In general molecules with PSA > 140 are poor in bioavailability; thus, 4e possesses limited oral bioavailability. The CYP2D6 value predicts the inhibitory and non-inhibitory aspect of **4e** on Cytochrome P450 2D6 enzyme. Compound 4e is predicted to be an inhibitor for CYP2D6; thus, drug–drug interactions and the side effects such as liver dysfunction are expected upon administration.

Based on the aforementioned results, bilosomes were proposed as an attempt to resolve the pitfalls that restrict the bioavailability, biological activity and cytotoxic activity of **4e**. Bile salt stabilized nanovesicular systems are established via the incorporation of bile salts into the lipid bilayers of the ordinary customized nano-vesicular systems [31]. The prime advantages behind the use of these bile salts in various pharmaceutical industries include intestinal penetration enhancers that induce the oral bioavailability of the drugs suffering from diminished aqueous solubility or/and intestinal permeability [32]. In addition, the advantages also include coating the surface of the vesicles by adopting proper polymeric coating such as polyethylene glycol (PEG) and establishing PEGylated bilosomes that are predisposed to diminishing systemic phagocytosis, resulting in an extension in systemic circulation duration in addition to the decline in toxicity profiles and elevating drug cellular uptake and cytotoxic activity [20]. Hence, the fabrication of **4e** as PEGylated bilosomes was assumed to promote its bioavailability at the tumor post, thus boosting its cytotoxic activity.

#### 2.3.2. Experimental Design, Fabrication and Statistical Evaluation of **4e** Pegylated Bilosomes

In an attempt to explore the influence of the formulation variables on the proposed responses, 2^3^ full factorial designs were implemented. Thus, the construction of eight experimental runs and their corresponding responses resulted in the following: EE%, PS and ZP prevailed in Table 2. The appropriate precision value of the model is used to investigate its suitability in order to maneuver the design [33]. A surpassing ratio of four is endorsed, which was perceived for all the dependent variables, as illustrated in Table 3. The adjusted and predicted R^2^ should be within approximately 0.20 of each other to represent reasonable agreement. As shown in Table 3, the predicted R2 values were in good harmony with the adjusted R2 in all dependent variables. Drug examination at different concentrations was conducted by using HPLC at λ_max_ 254 nm, exhibiting linear relationships between the drug concentration and peak area, which obeyed Beer–Lambert’s law (R^2^ = 0.9996) (Supplementary Materials Figure S1).

##### Influence of the Fabrication Variables on E.E%

**Figure 9:**
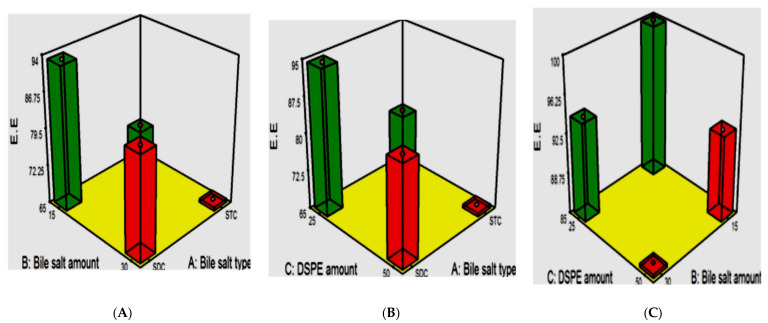
3D surface response plots exploit the impact of (**A**) bile salt type, (**B**) bile salt amount and (**C**) DSPE–mPEG-2000 amount on EE% of **4e**-loaded PEGylated bilosomes.

Ultimately the investigation of entrapment efficiency grants a clue on the power of the investigated vesicles in disclosing a prominent amount of **4e**. The percentage of **4e** disclosed in the vesicles ranged from 65.5 ± 2.3% to 100 ± 5.6%, as shown in Table 3. ANOVA results affirmed that the model with all variable bile salt type (A), bile salt amount (B) and DSPE–mPEG-2000 amount (C) significantly impacted (*p* = 0.0128) EE%, which is graphically illustrated in 3D surface plots of Figure 9.

Taking into consideration bile salt type (A), the formulae composed of SDC exploited significant (*p* = 0.005) higher drug entrapment compared to formulae consisting of STC. This can be clarified on the basis of lipophilicity and HLB values of the incorporated bile salts, where the lipophilicity of SDC (HLB 16) is higher than STC (HLB 22.1) [34]. This elevated lipophilicity proposed an expanded lipophilic core of the vesicles for the lipophilic drug **4e** to be disclosed in it [35].

ANOVA results declared that elevating the amount of bile salt (B) from 15 to 30 mg predisposed it to a prominent contrary impact on EE%. It was previously illustrated that the bile salts were capable of being assembled as helical mixed micelles in bilayers of the vesicles upon increasing its amount [36]. The increased bile salt amount will increase the possibility of the existence of mixed micelles which, in turn, will elevate the solubility of the drug **4e** in the aqueous phase [35]. Furthermore, elevated amounts of bile salt predisposes it to increase the fluidity of the vesicular membranes, which makes it leakier; hence, EE% of **4e** is diminished [35].

Regarding the impact of DSPE amount (C) on EE%, it can be observed that increasing the DSPE–mPEG-2000 amount from 25 to 50 mg resulted in a subsequent decline in EE%. This might be related to the creation of highly porous membranes upon increasing the amount of DSPE–mPEG-2000 [35], resulting in lessened EE% values. In addition, the higher solubilization power of DSPE–mPEG-2000 upon increasing its amount will potentially increase the solubility of **4e**, resulting in less encapsulation efficiency [37].

##### PDI and the Influence of the Fabrication Variables on PS

**Figure 10:**
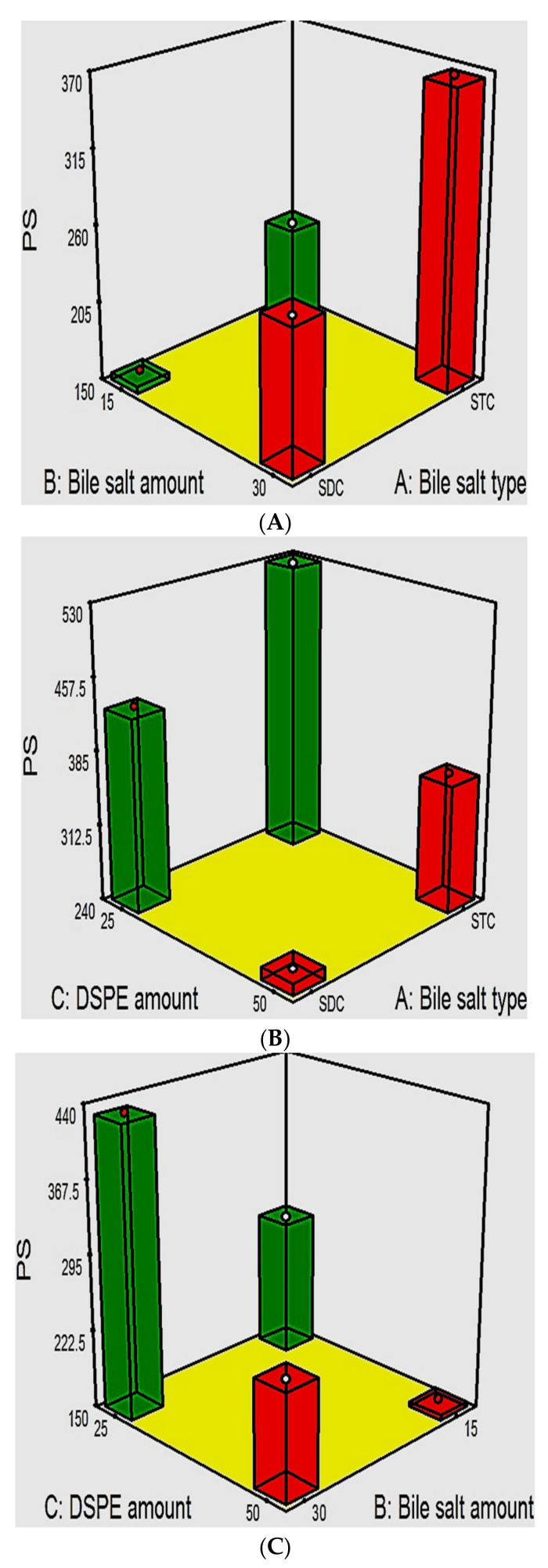
3D surface response plots exploit the impact of (**A**) Bile salt type, (**B**) Bile salt amount and (**C**) DSPE–mPEG-2000 amount on PS of **4e**-loaded PEGylated bilosomes.

PDI values figure out the level of monodispersity and the breadth of homogeneity: PDI values that proximate to zero denotes monodispersity, whilst the value that reaching one denotes polydispersity. The PDI values of **4e** loaded pegylated bilosomes as demonstrated in Table 3 ranged from 0.23 ± 0.05 to 0.565 ± 0.08. Therefore, the vesicles’ PDI values resides toward polydipersity but within a convenient range [38]. It is worth mentioning that the capabilities of the drug molecules to penetrate intestinal membrane and its fate in blood circulation are accredited to the particle size, where the minute particles exploit promoted intestinal penetration and extended drug retention time, hence enhanced biological activity. Table 3 shows that the average particle size of the investigated formulae ranged from 156.5 ± 18.2 to 527.2.1 ± 24.6 nm. ANOVA results confirmed that the model with all formulation variables bile salt type (A), bile salt amount (B) and DSPE–mPEG-2000 amount (C) had a prominent impact on PS (*p* = 0.0323), which is graphically displayed in 3D surface plots (Figure 10).

Regarding the impact of bile salt type (A), the PS of formulae fabricated using STC was massively higher (*p* = 0.0297) than those fabricated using SDC. This could be justified by the bile salt of lower HLB exhibiting the potential to develope tiny vesicles due to the decline in the vesicular surface free energy [39]. Aburahma [24] demonstrated that the bile salt of higher molecular weight will be the bulkier, hence increasing PS. The variation in molecular weight of both STC (537.68 gm/mol) and SDC (414.6 gm/mol) can be considered one of the reasons behind the higher PS of STC formulae [24].

Stunningly, elevation in system viscosity can be observed when increasing molecular weight, subsequently predisposing it to vesicular aggregations and expansion of vesicular size [35].

On another hand, raising the amount of bile salt from 15 mg to 30 mg predisposes it to suppression in PS (*p* = 0.0169), and this may be due to the decline in vesicular surface tension [24]. In addition, it was previously illustrated that increasing the amount of bile salt results in the creation of mixed micelles that exploit increasingly diminished sizes compared to that of the formulated vesicles [35].

Increasing the amount of DSPE–mPEG-2000 from 25 mg to 50mg predisposes it to a significant (*p* = 0.0169) decline in PS, where it is proclaimed previously that the elevation in PEG content permits a reasonable degree of steric hindrance resulting in the suppression of vesicular settling down and agglomeration, hence prohibiting vesicular aggregation and an increase in vesicular PS slows down the rate of vesicles’ precipitation and, hence, hinders vesicles’ agglomeration [37].

##### The Influence of the Compounding Variables on ZP

**Figure 11:**
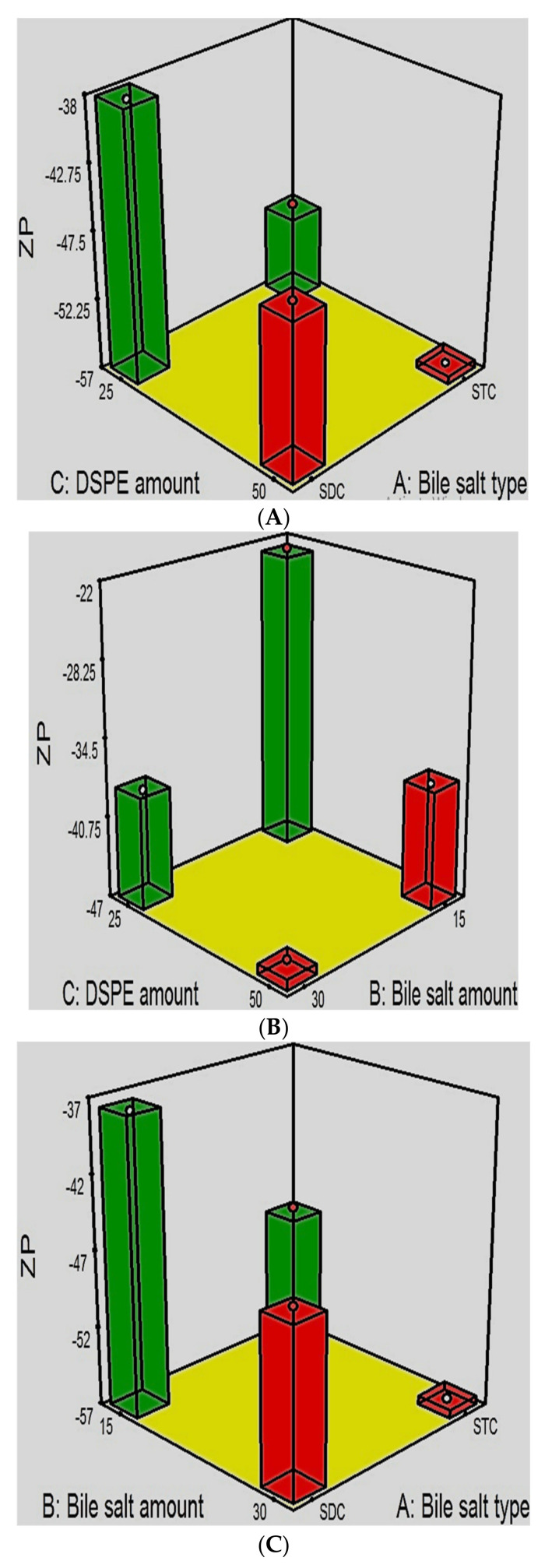
3D surface response plots exploit the impact of (**A**) bile salt type, (**B**) bile salt amount and (**C**) DSPE–mPEG-2000 amount on ZP of **4e**-loaded PEGylated bilosomes.

Zeta potential (ZP) offers a clue utilized in the appraisal of the extent of the vesicular system stability as it investigates the ultimate charges on the surface of the vesicles. Basically, ZP values around ± 30 Mv stands for the stability of the system, and this can be anticipated to be the confirmed electric repulsion between the vesicles [31]. In the conducted experiment, the assessed ZP values that form the prepared **4e** loaded PEGylated bilosomes ranged from −22.5 ± 3. 4 to −56.7 ± 7.4 mV (Table 3). ANOVA results showed that the model with all incorporated variables bile salt type (A), bile salt amount (B) and DSPE–mPEG-2000 (C) significantly influenced ZP (*p* = 0.0449), which is graphically illustrated in 3D surface plots (Figure 11).

ANOVA results revealed that by changing the type of bile salt (A), the formulae composed of STC exploited significant higher electronegativity (*p* = 0.0261) than those composed of SDC, and this may be attributed to the difference in the number of OH groups in the two incorporated bile salts as STC bears three hydroxyl group while SDC bears two hydroxyl groups [40]. In addition, the allocation of extensively charged taurine amino acid conjugated in the bile salt raised the negativity of ZP [35]. Moreover, the difference in molecular weight between the two bile salts (STC > SDC), as previously discussed, predisposes it to extensive accumulation of negative charges on the vesicular surface with the bulkier bile salt, as in case of STC [36].

Concerning amount of bile salt (B), the negative ZP values significantly (*p* = 0.0253) enlarged upon increasing the bile salts’ concentration from 15 mg to 30 mg due to the anionic character of the incorporated bile salts acquired by the presence of negatively charged groups in their structures [35].

Moreover, increasing the amount of DSPE–mPEG-2000 (C) from 25 mg to 50 mg results in significant (*p* = 0.0296) elevation in ZP values. This may be attributed to the creation of the electro negative layer of DSPE–mPEG on the vesicular surface, as DSPE–mPEG is characterized by its anionic nature acquired from the negatively charged functional groups in its structure [21]. These results are in accordance to Muthu et al., who revealed that the PEG coating results in an excessive negative charge on the coated vesicular surface compared to uncoated vesicles [21].

#### 2.3.3. Statistical Optimization and Validation of the Optimal **4e**-Loaded PEGylated Bilosomes

**Figure 12:**
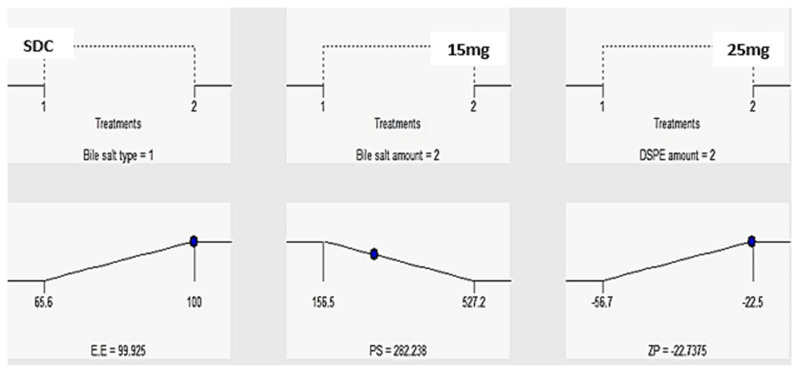
Optimization ramps for the studied independent variables exploit the optimum criteria of the formulation variables for **4e**-PEGylated bilosomal formulae with the predicted value of each measured formulation parameter.

Based on the precursive results, the optimal formula was elected by using Design expert software after the analysis of the results of the dependent variables. F7 composed of SDC as bile salt (amount of bile salt = 15 mg; amount of DSPE–mPEG-2000 = 25 mg) was found to be the optimal formula with a desirability value 0.868. In addition, the validity of our models was assessed by investigation of the percentage of discrepancy between the predicted and observed values concerning %EE, PS and ZP. As elicited from Table 3, the small percent of discrepancy as an absolute value (less than 10%) convinces the appropriateness of the statistical design to data analysis [41]. Figure 12 reveals the optimum criteria for **4e**-PEGylated bilosomal formulation for emulation.

#### 2.3.4. In Vitro Investigation of the Optimized **4e**-Loaded PEGylated Bilosome

##### Differential Scanning Calorimetry (DSC)

**Figure 13:**
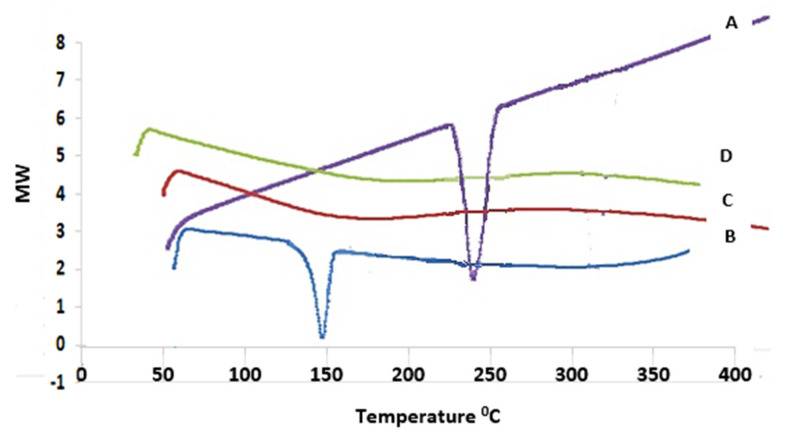
DSC thermograms: A: thermogram of pure **4e**; B: thermogram of cholesterol; C: thermogram of plain formula (F7); and D: thermogram of the optimum **4e**-loaded PEGylated bilosome (F7).

Figure 13 revealed the DSC thermograms of pure **4e** compound, cholesterol, plain lyophilized formula and the optimal lyophilized **4e**-loaded PEGylated bilosome (F7). DSC thermogram of pure 4e conveyed an acute pointed distinctive endotherm at approximately 227.4 °C, which is equivalent to its melting point, while cholesterol exhibited an endothemic peak at 148.5 °C corresponding to its melting point. DSC thermogram of lyophilized plain and **4e**-loaded PEGylated bilosome (F7) showed no characteristic peak of **4e** when assuming the complete remodeling of **4e** and other components as span 60, DSPE–mPEG-2000 and cholesterol from a crystalline to amorphous form, proposing good encapsulation of the drug within the vesicles.

##### Transmission Electron Microscope TEM

**Figure 14:**
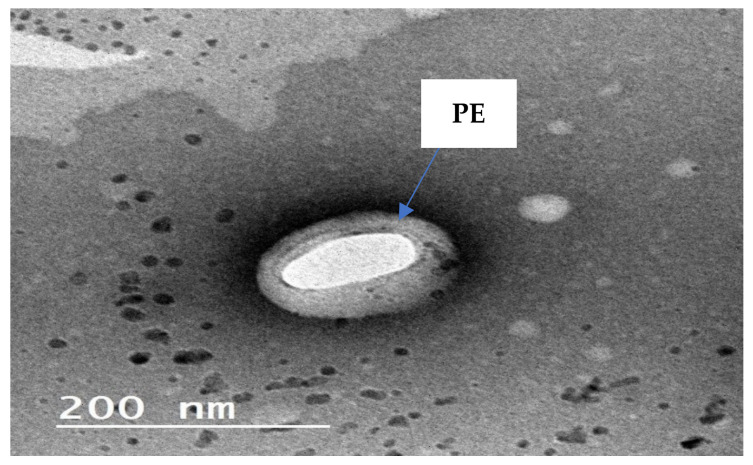
TEM of the elected optimized PEGylated bilosome (F7).

The configuration of TEM that is obviously emerged in Figure 14 assured that the bilosomal vesicles were spherical multilamellar vesicles (MLVs), and no vesicles with abnormal shapes were obvious. In addition, TEM image manifested that the vesicles had a smooth surface within the vicinity of PEG (faded colored fringe [42] surrounding the vesicles devoided of any drug crystal conforming the complete conformation of the drug into amorphous configuration, and it came in accordance to the results of DSC).

##### In Vitro Drug Release of Optimal Formula (F7) Correlated to **4e** Suspension

**Figure 15:**
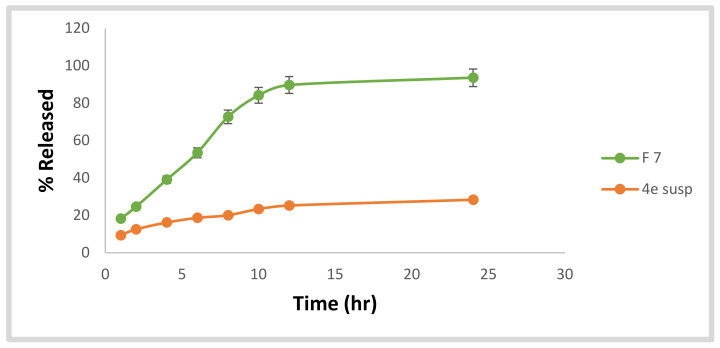
Percentage of **4e** released ± S.D. from the optimized PEGylated bilosome formula (F7) compared to that of **4e** suspension.

The manipulation of the in vitro release profile of drug loaded pegylated bilosome is in accordance to the extent of drug solubilization, stability and drug release configuration. Figure 15 revealed the gradual release of the drug from the optimized formula (F7) at 24 h, where the cumulated released amount of drug from F7 was 95.72 ± 3.82% compared to 24.35 ± 2.24% for the **4e** suspension (*p* < 0.05). This can be accredited to their characteristic as a colloidal particulate carriers pretending to be a drug pool [34]. Furthermore, (F7) release pattern primarily characterized by burst phases consequently controlled the drug release phase. Thus, 4e-loaded PEGylated bilosome was assumed to serve as stabilized nano-vesicles for extended period of time and guided the rise in agglomeration of **4e** at the tumor location [43]. These results may be accredited to the PEGylated layer that surrounds the vesicular surface, resulting in higher drug solubilization and increased rate of drug releases, and this due to the impact of hydrophilic and solubilization power of PEG to the drug [37].

##### Cytotoxic Activity of the Optimized Formula (F7) Compared to Target Compound **4e**

**Figure 16:**
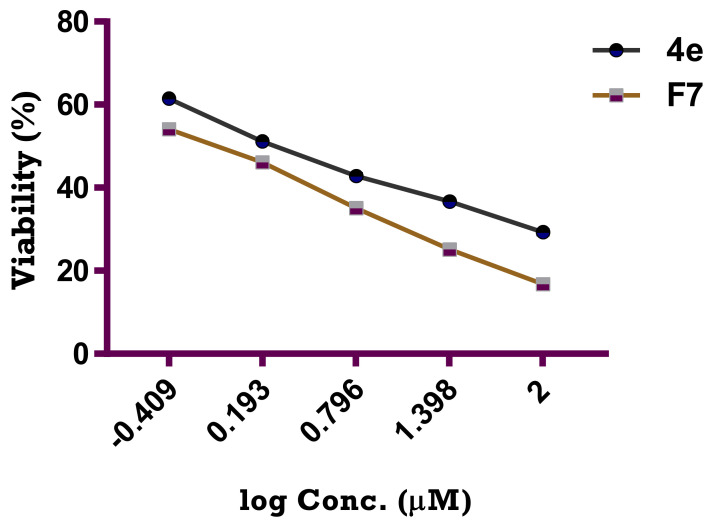
Graphical representation for comparison showing the effect of compounds **4e** and **4e**-loaded in PEGylated bilosomal formula (**F7**) at varying concentrations (μM) on MCF-7 cell line.

Cytotoxic activity against breast cancer MCF-7 cell line using MTT colorimetric assay was conducted for compound **4e** in its pure form correlated to optimal **4e**-loaded in the PEGylated bilosomal formula (F7), where the acquired IC_50_ values were 2.11 μM for pure **4e** and was significantly (*p* < 0.05) diminished at 0.75 ± 0.03 μM (Figure 16). It is worthy to mention that according to the characteristics of **4e** as being lipophilic in nature (Consensus LogPo/w = 3.56) with diminished aqueous solubility, it assumed to exploit an extensive suppression in its oral bioavailability [44]. The inclusion of **4e** in PEGylated bilosomal nano vesicles was very beneficial, and this was accredited to the significant improvement in **4e** solubility; thus, pegylation was approved to be utilized as solubility and absorption enhancers as well as for improving cellular uptake and cytotoxic activity on targeted cancer cells and elongation of drug circulation time up to more than 24 h [43]. Thus, F7 exhibits boosted cytotoxic efficacy than compared to the **4e** pure form alone, and this is in accordance to many studies that rely on PEGylated nano-vesicles in increasing the cytotoxic effect of the anticancer drugs towards the cancer cells [21,45].

## 3. Material and Methods

### 3.1. Material

Melting points were determined on open glass capillaries using Gallenkamp apparatus and are uncorrected. Infrared spectra were measured on a Shimadzu FT-IR 8400s infrared spectrophotometer (cm^−1^) using KBr disks, Faculty of Pharmacy, Cairo University, Cairo, Egypt. ^1^H and ^13^C-NMR spectra were determined in DMSO-*d_6_* with Bruker Avance-400 spectrometer operating at 400 MHz and 100 MHz, respectively, at Applied Nucleic Acid Research Centre (ANARC), Faculty of Science, Zagazig University, Zagazig, Egypt. TLC was performed on 2.5 × 5 cm aluminum plates coated with silicagel 60F-254 (*Merk,* Germany) in an appropriate solvent. Span 60 was purchased from Sigma-Aldrich Chemical Co. (St. Louis, MO, USA). Sodium taurocholate (STC) and sodium deoxycholate (SDC) were purchased from BASF Co. (Florham Park, NJ, USA). 1,2-distearoyl-sn-glycero-3-phosphoethanolamine-N [methoxy(polyethylene glycol)-2000] (DSPE–mPEG-2000) was a generous gift from Lipoid GmbH (Ludwigshafen, Germany). Cholesterol was obtained from ITX Biomedicals (Santa Ana, CA, USA). Sodium hydroxide, potassium dihydrogen orthophosphate and absolute ethanol were purchased from El-Nasr Chemical Co., Cairo, Egypt. The Spectra/PoreVR dialysis membrane (12,000–14,000 molecular weight cut off) was purchased from Spectrum Laboratories Inc., Los Angeles, CA, USA. All chemicals and solvents were of HPLC grade and were used as received.

### 3.2. Chemistry

#### 3.2.1. General Procedure for the Synthesis of 4-(Furan-2-ylmethylene)-2-(3,4,5-trimethoxyphenyl)oxazol-5(4*H*)-one (**1**)

A mixture of furfural (0.01 mol), 2-(3,4,5-trimethoxybenzamide)acetic acid (0.01 mol), freshly fused sodium acetate (0.01 mol) and acetic anhydride (0.03 mol), was placed in a 500 mL conical flask, and the reaction mixture was heated for about 3 h. Moreover, ethanol (50 mL) was added slowly to the reaction mixture, and the mixture was left overnight in a refrigerator. The crystalline product that was obtained was filtered off, washed with ice-cold ethanol (30 mL) and dried. Recrystallization from 1,4-dioxane was conducted in order to obtain compound **1**.

Yellow powder (2.67 g, 81.18%); m.p 183–185 °C; IR (KBr, *ν*_max_ cm^−1^): 3071, 3052 (arom.CH), 2897 (aliph.CH), 1803 (C=O lactone), 1606, 1593 (C=C). ^1^H-NMR (400 MHz, DMSO-*d_6_,* δ ppm): 3.80 (s, 3H, OCH_3_), 3.92 (s, 6H, 2OCH_3_), 6.77–6.93 (m, 1H, furan CH), 7.21 (s, 1H, olefinic CH), 7.37 (s, 2H, arom. CH), 7.66 (d, *J* = 3.6 Hz, 1H, furan CH), 8.00 – 8.15 (m, 1H, furan CH). ^13^C-NMR (100 MHz, DMSO-*d_6,_* δ ppm): 56.20 (2OCH_3_), 60.35 (OCH_3_), 105.18 (C2,6 trimethoxyphenyl), 114.25 (C3 furan), 117.11 (C4 furan), 120.05 (C1 trimethoxyphenyl), 120.52 (olefinic CH), 130.03 (olefinic CH), 142.20 (C4 trimethoxyphenyl), 147.93 (C5 furan), 149.87 (C2 furan), 153.28 (C3,5 trimethoxyphenyl), 161.94 (C2 oxazolone), 166.50 (C=O oxazolone). Analytically calculated for C_17_H_15_NO_6_ (329.30). Calculated: C, 62.00; H, 4.59; N, 4.25. Found: C, 61.95; H, 4.68; N, 4.33.

#### 3.2.2. General Procedure for the Synthesis of Ethyl 3-(Furan-2-yl)-2-(3,4,5-trimethoxybenzamido)acrylate (**2**)

A mixture of oxazolone **1** (0.005 mol), Et_3_N (0.005 mol) in absolute ethanol (30 mL) was heated under reflux for 1 h. After completion of the reaction, the reaction mixture was cooled and poured into ice cold water and left to stand at room temperature for an additional 1 h. The solid product was collected, washed with water and dried to produce compound **2**, which was purified by crystallization from absolute ethanol.

White powder (1.37 g, 73.19%); m.p 126–128 °C; IR (KBr, *ν*_max_ cm^−1^): 3470 (NH), 3071, 3052 (arom.CH), 2897 (aliph.CH), 1756, 1692 (C=O), 1606, 1593 (C=C). ^1^H-NMR (400 MHz, DMSO-*d_6_,* δ ppm): 1.23 (t, 3H, OCH_2_CH_3_), 3.89 (s, 3H, OCH_3_), 3.91 (s, 6H, 2OCH_3_), 4.19 (q, 2H, OCH_2_CH_3_), 6.63 (dd, *J* = 3.4, 1.7 Hz, 1H, furan CH), 6.87 (d, *J* = 3.4 Hz, 1H, furan CH), 7.25 (s, 1H, olefinic CH), 7.34 (s, 2H, arom. CH), 7.86 (d, *J* = 1.4 Hz, 1H, furan CH), 9.89 (s, 1H, NH, D_2_O exchange). ^13^C-NMR (100 MHz, DMSO-*d_6,_* δ ppm): 14.14 (CH_3_), 56.05 (2OCH_3_), 60.10 (OCH_3_), 60.86 (CH_2_), 105.27 (C2,6 trimethoxyphenyl), 112.58 (C3 ethyl acrylate), 115.91 (C3 furan), 120.81 (C4 furan), 123.72 (C2 ethyl acrylate), 128.51 (C1 trimethoxyphenyl), 140.47 (C4 trimethoxyphenyl), 145.59 (C5 furan), 149.17 (C2 furan), 152.71 (C3,5 trimethoxyphenyl), 164.47 (C1 ethyl acrylate), 164.96 (C=O). Analytically calculated for C_19_H_21_NO_7_ (375.37). Calculated: C, 60.79; H, 5.64; N, 3.73. Found: C, 61.05; H, 5.72; N, 3.55.

#### 3.2.3. General Procedure for the Synthesis of *N*-[1-(Furan-2-yl)-3-(isopropylamino)-3-oxoprop-1-en-2-yl]-3,4,5-trimethoxybenzamide (**3**)

A mixture of 4-(furan-2-ylmethylene)-2-(3,4,5-trimethoxyphenyl)oxazol-5(4*H*)-one (**1**) (0.005 mol) and isopropylamine (0.005 mol) in ethanol (30 mL) was refluxed for 1 h. The crystals that separated upon cooling the reaction mixture were collected and recrystallized from absolute ethanol to produce compound **3**.

White powder (1.42 g, 72.90%); m.p 168–170 °C; IR (KBr, *ν*_max_ cm^−1^): 3470 (NH), 3071, 3052 (arom.CH), 2897 (aliph.CH), 1756, 1692 (C=O), 1606, 1593 (C=C). ^1^H-NMR (400 MHz, DMSO-*d_6_,* δ ppm): 1.12 (d, *J* = 6.6 Hz, 6H, 2CH_3_), 3.74 (s, 3H, OCH_3_), 3.86 (s, 6H, 2OCH_3_), 3.94–4.06 (m, 1H, methylidene CH), 6.57 (dd, *J* = 3.4, 1.8 Hz, 1H, furan CH), 6.66 (d, *J* = 3.4 Hz, 1H, furan CH), 7.03 (s, 1H, olefinic CH), 7.38 (s, 2H, arom. CH), 7.75 (d, *J* = 1.4 Hz, 1H), 7.87 (s, 1H, NH, D_2_O exchange), 9.67 (s, 1H, NH, D_2_O exchange). ^13^C-NMR (100 MHz, DMSO-*d_6,_* δ ppm): 22.23 (2CH_3_), 40.97 (methylidene CH), 56.07 (2OCH_3_), 60.10 (OCH_3_), 105.49 (C2,6 trimethoxybenzamide), 112.19 (olefinic CH), 113.36 (C3 furan), 116.27 (C4 furan), 128.06 (C1 trimethoxybenzamide), 128.96 (olefinic CH), 140.33(C4 trimethoxybenzamide), 144.22 (C5 furan), 149.85 (C2 furan), 152.58 (C3,5 trimethoxybenzamide), 163.58 (C=O), 164.75 (C=O trimethoxybenzamide). Analytically calculated for C_20_H_24_N_2_O_6_ (388.41). Calculated: C, 61.84; H, 6.23; N, 7.21. Found: C, 61.62; H, 6.35; N, 7.26.

#### 3.2.4. General Procedure for the Synthesis of *N*-[1-(Furan-2-yl)-3-oxo-3-(arylamino)prop-1-en-2-yl]-3,4,5-trimethoxybenzamide **4a**–**e**

A mixture of 4-(furan-2-ylmethylene)-2-(3,4,5-trimethoxyphenyl)oxazol-5(4*H*)-one (**1**) (0.005 mol) and the appropriate aromatic amines (0.005 mol) in absolute ethanol (25 mL) was refluxed for 3–4 h. The crystals that separated upon cooling the reaction mixture were collected and recrystallized from absolute ethanol to produce products **4a**–**e**.

##### *N*-[1-(Furan-2-yl)-3-oxo-3-(o-tolylamino)prop-1-en-2-yl]-3,4,5-trimethoxybenzamide (**4a**)

White crystals (1.82 g, 83.48%); m.p 222–224 °C; IR (KBr, *ν*_max_ cm^−1^): 3470 (NH), 3071, 3052 (arom.CH), 2897 (aliph.CH), 1756, 1692 (C=O), 1606, 1593 (C=C). ^1^H-NMR (400 MHz, DMSO-*d_6_,* δ ppm): 2.51 (s, 3H, CH_3_), 3.78 (s, 3H, OCH_3_), 3.89 (s, 6H, 2OCH_3_), 6.85 (dd, *J* = 3.4, 1.4 Hz, 1H, furan CH), 7.06 (d, *J* = 3.4, 1H, furan CH), 7.20 (s, 1H, olefinic CH), 7.31 (s, 2H, arom. CH), 7.36–7.39 (m, 2H, arom. CH), 7.59 (d, *J* = 3.4 Hz, 1H, arom. CH), 7.66 (d, *J* = 3.5 Hz, 1H, arom. CH), 8.07–8.12 (m, 1H, furan CH), 8.30 (s, 1H, NH, D_2_O exchange), 10.18 (s, 1H, NH, D_2_O exchange). ^13^C-NMR (100 MHz, DMSO-*d_6,_* δ ppm): 21.05 (CH_3_), 56.04 (2OCH_3_), 60.28 (OCH_3_), 105.62 (C2,6 trimethoxybenzamide), 114.08 (olefinic CH),114.24 (C3 furan),114.45 (C4 furan), 117.10 (C6 tolyl), 119.40 (C5 tolyl), 120.04 (C1 trimethoxybenzamide), 122.27 (C4 tolyl), 130.03 (C3 tolyl), 133.42 (C2 tolyl), 140.94 (olefinic CH), 147.09 (C1 tolyl), 147.91 (C4 trimethoxybenzamide), 149.87 (C5 furan), 152.81 (C3,5 trimethoxybenzamide), 157.30 (C5 furan), 166.50 (C=O trimethoxybenzamide), 167.70 (C=O amide). Analytically calculated for C_24_H_24_N_2_O_6_ (436.46). Calculated: C, 66.04; H, 5.54; N, 6.42. Found: C, 66.13; H, 5.61; N, 6.36.

##### *N*-[1-(Furan-2-yl)-3-oxo-3-(m-tolylamino)prop-1-en-2-yl]-3,4,5-trimethoxybenzamide (**4b**)

White crystals (1.92 g, 88.07%); m.p 217–219 °C; IR (KBr, *ν*_max_ cm^−1^): 3470 (NH), 3071, 3052 (arom.CH), 2897 (aliph.CH), 1756, 1692 (C=O), 1606, 1593 (C=C). ^1^H-NMR (400 MHz, DMSO-*d_6_,* δ ppm): 2.28 (s, 3H, CH_3_), 3.74 (s, 3H, OCH_3_), 3.87 (s, 6H, 2OCH_3_), 6.61 (dd, *J* = 3.3, 1.7 Hz, 1H, arom. CH), 6.77 (d, *J* = 3.4 Hz, 1H, furan CH), 6.89 (d, *J* = 7.5 Hz, 1H, arom. CH), 7.10 (s, 1H, olefinic CH), 7.19 (t, *J* = 7.8 Hz, 1H, arom. CH), 7.42 (s, 2H, arom. CH), 7.49–7.59 (m, 2H, furan and arom. CH), 7.77–7.85 (m, 1H, furan CH), 10.06 (s, 1H, NH, D_2_O exchange), 10.15 (s, 1H, NH, D_2_O exchange). ^13^C-NMR (100 MHz, DMSO-*d_6,_* δ ppm): 21.19 (CH_3_), 56.06 (2OCH_3_), 60.12 (OCH_3_), 105.57 (C2,6 trimethoxybenzamide), 112.35 (olefinic CH), 113.98 (C3 furan), 116.69 (C4 furan), 117.35 (C6 tolyl), 120.68 (C5 tolyl), 124.10 (C1 trimethoxybenzamide), 128.32 (C4 tolyl), 128.82 (olefinic CH), 137.60 (C2 tolyl), 139.14 (C1 tolyl), 140.40 (C4 trimethoxybenzamide), 144.54 (C5 furan), 149.77 (C2 furan), 152.61 (C3,5 trimethoxybenzamide), 163.57 (C=O trimethoxybenzamide), 165.12 (C=O amide). Analytically calculated for C_24_H_24_N_2_O_6_ (436.46). Calculated: C, 66.04; H, 5.54; N, 6.42. Found: C, 66.10; H, 5.47; N, 6.39.

##### *N*-[1-(Furan-2-yl)-3-(4-methoxyphenylamino)-3-oxoprop-1-en-2-yl]-3,4,5-trimethoxybenzamide (**4c**)

Pale yellow crystals (1.78 g, 78.59%); m.p 249–251 °C; IR (KBr, *ν*_max_ cm^−1^): 3470 (NH), 3071, 3052 (arom.CH), 2897 (aliph.CH), 1756, 1692 (C=O), 1606, 1593 (C=C). ^1^H-NMR (400 MHz, DMSO-*d_6_,* δ ppm): 3.73 (s, 3H, OCH_3_), 3.74 (s, 3H, OCH_3_), 3.87 (s, 6H, 2OCH_3_), 6.61 (dd, *J* = 3.3, 1.8 Hz, 1H, furan CH), 6.75 (d, *J* = 3.4 Hz, 1H, furan CH), 6.82–6.95 (d, *J* = 9.1 Hz, 2H, arom. CH), 7.11 (s, 1H, olefinic CH), 7.41 (s, 2H, arom. CH), 7.62 (d, *J* = 9.1 Hz, 2H, arom. CH), 7.80 (d, *J* = 1.3 Hz, 1H, furan CH), 9.94 (s, 2H, 2NH, D_2_O exchange). ^13^C-NMR (100 MHz, DMSO-*d_6,_* δ ppm): 55.16 (OCH_3_), 56.07 (2OCH_3_), 60.12 (OCH_3_), 105.57 (C2,6 trimethoxybenzamide), 112.33 (olefinic CH), 113.61 (C3,5 methoxyphenyl), 113.61 (C3 furan), 116.58 (C4 furan), 121.78 (C2,6 methoxyphenyl), 128.22 (C1 trimethoxybenzamide), 128.84 (C1 methoxyphenyl), 132.26 (olefinic CH), 140.40 (C4 trimethoxybenzamide), 144.52 (C5 furan), 149.79 (C2 furan), 152.61 (C3,5 trimethoxybenzamide), 155.40 (C4 methoxyphenyl), 163.15 (C=O trimethoxybenzamide), 165.09 (C=O amide). Analytically calculated for C_24_H_24_N_2_O_7_ (452.46). Calculated: C, 63.71; H, 5.35; N, 6.19. Found: C, 63.88; H, 5.27; N, 6.24.

##### *N*-[1-(Furan-2-yl)-3-(4-hydroxyphenylamino)-3-oxoprop-1-en-2-yl]-3,4,5-trimethoxybenzamide (**4d**)

White crystals (1.65 g, 75.17%); m.p 214–216 °C; IR (KBr, *ν*_max_ cm^−1^): 3470 (NH), 3071, 3052 (arom.CH), 2897 (aliph.CH), 1756, 1692 (C=O), 1606, 1593 (C=C). ^1^H-NMR (400 MHz, DMSO-*d_6_,* δ ppm): 3.74 (s, 3H, OCH_3_), 3.86 (s, 6H, 2OCH_3_), 6.60 (dd, *J* = 3.2, 1.7 Hz, 1H, furan CH), 6.71 (d, *J* = 8.8 Hz, 2H, arom. CH), 6.74 (d, *J* = 3.4 Hz, 1H, furan CH), 7.10 (s, 1H, olefinic CH), 7.41 (s, 2H, arom. CH), 7.47 (d, *J* = 8.8 Hz, 2H, arom. CH), 7.73–7.88 (m, 1H, furan CH), 9.83 (s, 1H, NH, D_2_O exchange), 9.97 (s, 1H, NH, D_2_O exchange). ^13^C-NMR (100 MHz, DMSO-*d_6,_* δ ppm): 56.09 (2OCH_3_), 60.14 (OCH_3_), 105.59 (C2,6 trimethoxybenzamide), 112.33 (olefinic CH), 113.61 (C3 furan), 114.89 (C3,5 hydroxyphenyl), 116.56 (C4 furan), 122.07 (C2,6 hydroxyphenyl), 128.34 (C1 trimethoxybenzamide), 128.89 (C1 hydroxyphenyl), 130.70 (olefinic CH), 140.41 (C4 trimethoxybenzamide), 144.48 (C5 furan), 149.84 (C2 furan), 152.62 (C3,5 trimethoxybenzamide), 153.62 (C4 hydroxyphenyl), 163.00 (C=O trimethoxybenzamide), 165.13 (C=O amide). Analytically calculated for C_23_H_22_N_2_O_7_ (438.43). Calculated: C, 63.01; H, 5.06; N, 6.39. Found: C, 62.91; H, 5.12; N, 6.47.

##### *N*-[1-(Furan-2-yl)-3-(3-hydroxy-4-methoxyphenylamino)-3-oxoprop-1-en-2-yl]-3,4,5-trimethoxybenzamide (**4e**)

Pale yellow crystals (1.90 g, 81.19%); m.p 226–228 °C; IR (KBr, *ν*_max_ cm^−1^): 3470 (NH), 3071, 3052 (arom.CH), 2897 (aliph.CH), 1756, 1692 (C=O), 1606, 1593 (C=C). ^1^H-NMR (400 MHz, DMSO-*d_6_,* δ ppm): 33.73 (s, 3H, OCH_3_), 3.74 (s, 3H, OCH_3_), 3.87 (s, 6H, 2OCH_3_), 6.60 (dd, *J* = 3.3, 1.8 Hz, 1H, furan CH), 6.75 (d, *J* = 3.4 Hz, 1H, furan CH), 6.84 (d, *J* = 8.8 Hz, 1H, arom. CH), 7.04–7.10 (m, 2H, arom. and olefinic CH), 7.28 (d, *J* = 2.4 Hz, 1H, arom. CH), 7.41 (s, 2H, arom. CH), 7.79 (d, *J* = 1.3 Hz, 1H, furan CH), 9.81 (s, 1H, NH, D_2_O exchange), 9.93 (s, 1H, NH, D_2_O exchange). ^13^C-NMR (100 MHz, DMSO-*d_6,_* δ ppm): 55.87 (OCH_3_), 56.08 (2OCH_3_), 60.13 (OCH_3_), 105.56 (C2,6 trimethoxybenzamide), 108.63 (C2 hydroxymethoxyphenyl), 111.14 (olefinic CH), 112.18 (C3 furan), 112.32 (C4 furan), 113.87 (C5 hydroxymethoxyphenyl), 116.49 (C6 hydroxymethoxyphenyl), 128.31 (C1 trimethoxybenzamide), 128.85 (C1 hydroxymethoxyphenyl), 132.69 (olefinic CH), 140.41 (C4 trimethoxybenzamide), 144.00 (C5 furan), 144.49 (C4 hydroxymethoxyphenyl), 146.20 (C3 hydroxymethoxyphenyl), 149.80 (C2 furan), 152.62 (C3,5 trimethoxybenzamide), 163.03 (C=O trimethoxybenzamide), 165.08 (C=O amide). Analytically calculated for C_24_H_24_N_2_O_8_ (468.46). Calculated: C, 61.53; H, 5.16; N, 5.98. Found: C, 61.61; H, 5.21; N, 5.91.

#### 3.2.5. General Procedure for the Synthesis of *N*-[3-(Arylamino)-1-(furan-2-yl)-3-oxoprop-1-en-2-yl]-3,4,5-trimethoxybenzamide **5a**–**d**

A mixture of oxazolone **1** (0.005 mol), substituted benzyl amines (0.005 mol) and anhydrous sodium acetate (0.006) in glacial acetic acid (30 mL) was heated under reflux for 1–2 h. The reaction mixture was cooled, and the resulting solid was filtered off, dried and crystallized from acetic acid/water to provide compounds **5a**–**d**.

##### *N*-[1-(Furan-2-yl)-3-((3-methylbenzyl)amino)-3-oxoprop-1-en-2-yl]-3,4,5-trimethoxybenzamide (**5a**)

White powder (1.74 g, 77.41%); m.p 175–177 °C; IR (KBr, *ν*_max_ cm^−1^): 3470 (NH), 3071, 3052 (arom.CH), 2897 (aliph.CH), 1756, 1692 (C=O), 1606, 1593 (C=C). ^1^H-NMR (400 MHz, DMSO-*d_6_,* δ ppm): 2.29 (s, 3H, CH_3_), 3.74 (s, 3H, OCH_3_), 3.87 (s, 6H, 2OCH_3_), 4.35 (d, *J* = 6.1 Hz, 2H, CH_2_NH), 6.59 (dd, *J* = 3.4, 1.8 Hz, 1H, furan CH), 6.72 (d, *J* = 3.4 Hz, 1H, furan CH), 7.04 (d, *J* = 7.4 Hz, 1H, arom. CH), 7.10 (d, *J* = 7.6 Hz, 1H, arom. CH), 7.13 (s, 1H, olefinic CH), 7.16–7.25 (m, 2H, arom. CH), 7.41 (s, 2H, arom. CH), 7.78 (d, *J* = 1.4 Hz, 1H, furan CH), 8.61 (t, *J* = 6.1 Hz, 1H, NH, D_2_O exchange), 9.80 (s, 1H, NH, D_2_O exchange). ^13^C-NMR (100 MHz, DMSO-*d_6,_* δ ppm): 21.05 (CH_3_), 42.55 (methylene CH_2_), 56.09 (2OCH_3_), 60.13 (OCH_3_), 105.61 (C2,6 trimethoxybenzamide), 112.31 (olefinic CH), 113.96 (C3 furan), 117.66 (C4 furan), 124.23 (C6 methylbenzyl), 127.18 (C4 methylbenzyl), 127.50 (C5 methylbenzyl), 127.81 (C1 trimethoxybenzamide), 128.04 (C2 methylbenzyl), 129.00 (olefinic CH), 137.12 (C1 methylbenzyl), 139.65 (C3 methylbenzyl), 140.38 (C4 trimethoxybenzamide), 144.57 (C5 furan), 149.79 (C2 furan), 152.58 (C3,5 trimethoxybenzamide), 164.39 (C=O amide), 165.22 (C=O trimethoxybenzamide). Analytically calculated for C_25_H_26_N_2_O_6_ (450.48): C, 66.65; H, 5.82; N, 6.22. Found: C, 66.92; H, 5.91; N, 6.29.

##### *N*-[1-(Furan-2-yl)-3-((2-methoxybenzyl)amino)-3-oxoprop-1-en-2-yl]-3,4,5-trimethoxybenzamide (**5b**)

White powder (1.67 g, 71.72%); m.p 166–168 °C; IR (KBr, *ν*_max_ cm^−1^): 3470 (NH), 3071, 3052 (arom.CH), 2897 (aliph.CH), 1756, 1692 (C=O), 1606, 1593 (C=C). ^1^H-NMR (400 MHz, DMSO-*d_6_,* δ ppm): 3.74 (s, 3H, OCH_3_), 3.79 (s, 3H, OCH_3_), 3.86 (s, 6H, 2OCH_3_), 4.34 (d, *J* = 6.0 Hz, 2H, CH_2_NH), 6.58 (dd, *J* = 3.4, 1.8 Hz, 1H, furan CH), 6.72 (d, *J* = 3.4 Hz, 1H, furan CH), 6.90 (t, *J* = 7.4 Hz, 1H, arom. CH), 6.96 (d, *J* = 7.8 Hz, 1H, arom. CH), 7.18–7.28 (m, 3H, olefinic and arom. CH), 7.40 (s, 2H, arom. CH), 7.77 (d, *J* = 1.4 Hz, 1H, furan CH), 8.43 (t, *J* = 6.1 Hz, 1H, NH, D_2_O exchange), 9.82 (s, 1H, NH, D_2_O exchange). ^13^C-NMR (100 MHz, DMSO-*d_6,_* δ ppm): 37.74 (methylene CH_2_), 55.27 (OCH_3_), 56.11 (2OCH_3_), 60.14 (OCH_3_), 105.60 (C2,6 trimethoxybenzamide), 110.21 (olefinic CH), 112.32 (C3 methoxybenzyl), 114.07 (C3 furan), 117.74 (C4 furan), 120.07 (C5 methoxybenzyl), 126.93 (C1 methoxybenzyl), 127.14 (C4 methoxybenzyl), 127.39 (C6 methoxybenzyl), 127.69 (C1 trimethoxybenzamide), 129.01 (olefinic CH), 140.39 (C4 trimethoxybenzamide), 144.62 (C5 furan), 149.78 (C2 furan), 152.59 (C3,5 trimethoxybenzamide), 156.25 (C2 methoxybenzyl), 164.46 (C=O amide), 165.36 (C=O trimethoxybenzamide). Analytically calculated for C_25_H_26_N_2_O_7_ (466.48): C, 64.37; H, 5.62; N, 6.01. Found: C, 64.29; H, 5.55; N, 6.09.

##### *N*-[1-(Furan-2-yl)-3-(3-methoxybenzylamino)-3-oxoprop-1-en-2-yl]-3,4,5-trimethoxybenzamide (**5c**)

White powder (1.83 g, 78.52%); m.p 164–166 °C; IR (KBr, *ν*_max_ cm^−1^): 3470 (NH), 3071, 3052 (arom.CH), 2897 (aliph.CH), 1756, 1692 (C=O), 1606, 1593 (C=C). ^1^H-NMR (400 MHz, DMSO-*d_6_,* δ ppm): 3.74 (s, 3H, OCH_3_), 3.76 (s, 3H, OCH_3_), 3.87 (s, 6H, 2OCH_3_), 4.37 (d, *J* = 6.1 Hz, 2H, CH_2_NH), 6.59 (dd, *J* = 3.3, 1.8 Hz, 1H, furan CH), 6.73 (d, *J* = 3.4 Hz, 1H, furan CH), 6.78 (dd, *J* = 8.1, 2.1 Hz, 1H, arom. CH), 6.88 (d, *J* = 7.6 Hz, 1H, arom. CH), 6.92 (s, 1H, olefinic CH), 7.20 (s, 1H, arom. CH), 7.23 (d, *J* = 7.8 Hz, 1H, arom. CH), 7.41 (s, 2H, arom. CH), 7.78 (s, 1H, furan CH), 8.64 (t, *J* = 6.1 Hz, NH, D_2_O exchange), 9.83 (s, 1H, NH, D_2_O exchange). ^13^C-NMR (100 MHz, DMSO-*d_6,_* δ ppm): 42.46 (methylene CH_2_), 54.95 (OCH_3_), 56.07 (2OCH_3_), 60.12 (OCH_3_), 105.59 (C2,6 trimethoxybenzamide), 112.18 (olefinic CH), 112.29 (C2 methoxybenzyl), 112.43 (C4 methoxybenzyl), 113.97 (C3 furan), 117.53 (C4 furan), 119.18 (C6 methoxybenzyl), 127.53 (C1 trimethoxybenzamide), 128.94 (C5 methoxybenzyl), 129.10 (olefinic CH), 140.38 (C1 methoxybenzyl), 141.41 (C4 trimethoxybenzamide), 144.57 (C5 furan), 149.77 (C1 furan), 152.56 (C3,5 trimethoxybenzamide), 159.30 (C3 methoxybenzyl), 164.38 (C=O), 165.25 (C=O trimethoxybenzamide). Analytically calucated for C_25_H_26_N_2_O_7_ (466.48): C, 64.37; H, 5.62; N, 6.01. Found: C, 64.47; H, 5.79; N, 6.93.

##### *N*-[3-((3,4-Dimethoxybenzyl)amino)-1-(furan-2-yl)-3-oxoprop-1-en-2-yl]-3,4,5-trimethoxybenzamide (**5d**)

White powder (1.69 g, 68.16%); m.p 124-126 °C; IR (KBr, *ν*_max_ cm^−1^): 3470 (NH), 3071, 3052 (arom.CH), 2897 (aliph.CH), 1756, 1692 (C=O), 1606, 1593 (C=C). ^1^H-NMR (400 MHz, DMSO-*d_6_,* δ ppm): 3.72 (s, 3H, OCH_3_), 3.74 (s, 3H, OCH_3_), 3.77 (s, 3H, OCH_3_), 3.87 (s, 6H, 2OCH_3_), 4.33 (d, *J* = 6.1 Hz, 2H, CH_2_NH), 6.59 (dd, *J* = 3.4, 1.8 Hz, 1H, furan CH), 6.72 (d, *J* = 3.4 Hz, 1H, furan CH), 6.82 (d, *J* = 8.2 Hz, 1H, arom. CH), 6.87 (s, 1H, olefinic CH), 6.97 (d, *J* = 1.9 Hz, 1H, arom. CH), 7.17 (s, 1H, arom. CH), 7.41 (s, 2H, arom. CH), 7.78 (s, 1H, furan CH), 8.58 (t, *J* = 6.1 Hz, 1H, NH, D_2_O exchange), 9.84 (s, 1H, NH, D_2_O exchange). ^13^C-NMR (100 MHz, DMSO-*d_6,_* δ ppm): 42.19 (methylene CH_2_), 55.35 (OCH_3_), 55.58 (OCH_3_), 56.06 (2OCH_3_), 60.11 (OCH_3_), 105.57 (C2,6 trimethoxybenzamide), 111.13 (C2 dimethoxybenzyl), 111.62 (olefinic CH), 112.27 (C5 dimethoxybenzyl), 113.87 (C3 furan), 117.27 (C4 furan), 119.01 (C6 dimethoxybenzyl), 127.71 (C1 trimethoxybenzamide), 128.88 (C1 dimethoxybenzyl), 132.24 (olefinic CH), 140.37 (C4 trimethoxybenzamide), 144.51 (C5 furan), 147.52 (C4 dimethoxybenzyl), 148.62 (C2 furan), 149.77 (C3 dimethoxybenzyl), 152.56 (C3,5 trimethoxybenzamide), 164.30 (C=O amide), 165.20 (C=O trimethoxybenzamide). Analytically calculated for C_26_H_28_N_2_O_8_ (496.51): C, 62.89; H, 5.68; N, 5.64. Found: C, 63.03; H, 5.62; N, 5.56.

### 3.3. Biological Studies

#### 3.3.1. Cytotoxic Activity against Breast MCF-7 Cancer Cell Line

Cytotoxic activity of the newly prepared acrylamide derivatives **2**–**5d** were carried out against breast MCF-7 cancer cell line using the MTT assay method. Compound **4e** was screened for its effects on normal breast cell line MCF-10A. Cells at density of 1 × 10^4^ were seeded in a 96-well plate at 37 °C for 48 h under 5% CO_2_. After incubation, the cells were treated with different concentrations of the test compounds and incubated for 24 h. After 24 h of drug treatment, 20 μL of MTT solution at 5 mg/mL was applied and incubated for 4 h at 37 °C. Dimethyl sulphoxide (DMSO) in volume of 100 µL was added to each well to dissolve the purple formazan that had formed. The color intensity of the formazan product, which represents the growth condition of the cells, is quantified by using an ELISA plate reader (EXL 800, USA) at 570 nm absorbance. The experimental conditions were carried out with at least three replicates, and the experiments were repeated at least three times.

#### 3.3.2. Tubulin Assays

Compound **4e** and Col were evaluated against tubulin polymerization inhibitory activity according to manufacturer’s instructions [26].

#### 3.3.3. DNA Flow Cytometry Analysis

##### Cell Cycle Analysis Compound **4e**

MCF-7 cells (2 × 10^5^/well) were harvested and washed twice in PBS. After that, the cells were incubated at 37 °C and 5% CO_2_. The medium was replaced with DMSO 1% *v*/*v* containing the tested compound **4e** (2.11 μM) and PTX (0.1 μM), then incubated for 48 h, washed twice in PBS, fixed with 70% ethanol, rinsed again with PBS and then stained with DNA fluorochrome PI for 15 min at 37 °C. The samples were analyzed by flow cytometry on a FACS Calibur flow cytometer (Becton and Dickinson, Heidelberg, Germany).

##### Annexin V FITC/PI Apoptosis Detection Staining Assay

Apoptotic cells death was investigated by using fluorescent Annexin V-FITC/ PI detection kit by flow cytometry assay. Briefly, MCF-7 cells (2 × 10^5^) after incubation for 12 h were used. Cells were treated with compound **4e** (2.11 μM) and PTX (0.1 μM) for 48 h, then the cells were harvested and stained with Annexin V-FITC/ PI dye for 15 min in the dark at 37 °C. The samples were analyzed by using FACS Calibur flow cytometer (Becton and Dickinson, Heidelberg, Germany).

#### 3.3.4. Caspase 3/7 Green Flow Cytometry Assay

The enzymatic activities of *c*aspase 3/7 in MCF-7 cell line was detected in the presence of compound **4e** (2.11 μM) and PTX (0.1 μM) using *c*aspase 3/7 green flow cytometry assay kit as per the manufacturer’s instructions. Briefly, PTX and compound **4e** treated MCF-7 cells (2.5 × 10^5^/mL) were washed with ice cold PBS, and cell lysates were prepared and combined with reaction buffer and incubated with specific colorimetric substrates (Caspase 3/7 Detection Reagent) at 37 °C for 6 h. The samples were analyzed at 488 nm in a BD FACS Calibur flow cytometer. All experiments were performed in triplicates.

#### 3.3.5. Molecular Docking Study

Molecular docking study was performed using MOE software program (MOE 2009.10). The tubulin crystal structure (PDB entry: 1SA0) was obtained from a protein data bank (Supplementary Materials).

### 3.4. Tailoring of **4e**-Loaded PEGylated Bilosome

Minute modifications were stuck to thin film hydration technique, which was manipulated for the development of 4e-loaded PEGylated bilosomes [21,23]. More precisely, (10 mL) a blend of chloroform and methanol (2:1) was incorporated to dissolve 4e (20 mg), span 60 (100 mg) and cholesterol (25 mg) with different amounts of DSPE–mPEG-2000 (25 mg or 50 mg) in a round bottom flask (Table 3). The acquired organic solution was dispelled at 60 °C under reduced pressure for 30 min by utilizing a rotary evaporator (Rotavapor, Heidolph VV 2000; Heidolph Instruments, Kehlheim, Germany) up until the formation of completely dry thin film. Formerly, the attained dry film was splashed using 10 mL phosphate buffer solution at 60 °C, enclosing different types of bile salts (SDC or STC) in different amounts (15 mg or 30 mg). In addition, the developed PEGylated bilosomal dispersions were exposed to sonication for 10 min in a bath sonicator (Ultra Sonicator, Model LC 60/H Elma, Germany) at room temperature aspiring for further suppression in particle size and stability. The attained formulae were kept at 4 °C for further characterization.

### 3.5. HPLC Investigation

Drug stock solution of 1 mg/mL in methanol was prepared, and a calibration curve was constructed utilizing six dilutions that were prepared in concentrations of 100, 200, 400, 600, 800 and 1000 µg/mL. All solutions were filtered using 0.22 µm syringe filter and then 10 µL was subjected to HPLC analysis using Waters-2690 Alliance^®^ HPLC system (WatersTM, Milford, MA, USA), and HPLC conditions were in mobile phase: water (50:50); flow rate: 1 mL/min [46]. A distinct peak of the drug was observed at 254 nm. Each experiment was conducted in triplicate, and the mean peak area was configured versus the drug concentration.

### 3.6. In Vitro Analysis and Optimization of **4e**-Loaded PEGylated Bilosomes

#### 3.6.1. Investigation of the Entrapment Efficiency Percentage (EE%)

In order to investigate the percentage of **4e** charged within the formulated PEGylated bilosomal dispersion precisely, 1ml of **4e**-loaded PEGylated bilosomal dispersion (resembling 2 mg of the drug) was diluted with 5 mL distilled water and manually agitated for 2 min. Cooling centrifugation technique for one hour was used to decouple the unembedded **4e** from **4e**-loaded PEGylated bilosome at 15,000 rpm and 4 °C (Beckman, Fullerton, NU, Canada) [22]. The sedimented vesicles were assembled away, rinsed twice with distilled water and centrifuged again for 30 min. The sonication of the separated particles using methanol was performed to predict the amount of the enclosed MH. The concentration of the embedded **4e** within the vesicles was allocated via HPLC at λmax 254 nm (EE%) and was calculated as follows.

% of **4e** entrapped = (Amount of **4e** disclosed/overall amount of **4e**) × 100 (1)

#### 3.6.2. Investigation of Zeta Potential, Vesicle Size and PDI

The tailored **4e**-loaded PEGylated bilosome droplet size, zeta potential and PDI were investigated by utilizing Malvern sizer (Malvern Instruments, Malvern, UK). The amount of 10 mL distilled water was utilized in order to dilute 0.1 mL of **4e**-loaded PEGylated bilosomal dispersion within a glass tube that was manipulated, and then it was convulsed manually for 5 min. The manipulated technique was the dynamic laser scattering technique used to determine the distribution size at 25 °C by using 45 mm focus lens and beam lengths of 2.4 mm. The test was performed in triplicate [23].

### 3.7. Conduction of Experimental Design and Selecting the Optimal **4e**-Loaded PEGylated Bilosome

A 2^3^ factorial experiment was developed to assess the impact of multiple factors in the fabrication of PEGylated bilosome via Design Expert^®^ software version 7 (Stat Ease, Inc., Minneapolis, MN, USA). The tailoring of 8 runs was yielded from the constructed design. Three factors were considered, bile salt type (A), bile salt amount (B) and DSPE–mPEG-2000 amount (C), to be independent variables, whereas EE% (Y1), PS (Y2), ZP (Y3) and Q8h (Y4) were picked as dependent variables. Moreover, based on the maximum EE%, ZP and minimum globule size, the optimum **4e**-loaded PEGylated bilosomal formula was primed. Statistical analysis of the data was performed by utilizing Design Expert^®^ 7 software. In addition, the statistical analysis conducted via ANOVA was implemented to highlight the prime impacts of the variables under exploration; the significance of each variable was analyzed, and the best formula with the superior desirability value was picked for further assessments [46].

### 3.8. In Vitro Investigation of the Optimum **4e**-Loaded PEGylated Bilosomal Formula

#### 3.8.1. Lyophilization of the Optimized PEGylated Bilosomal Formula

The solidification of the optimum **4e**-loaded PEGylated bilosomal formula was manipulated via lyophilization technique (Alpha 2–4, CHRIST, Osterodeam Harz, Germany), where mannitol (5% *w*/*v*) as lyoprotectant was utilized to disrupt the lysis of the vesicles. Accordingly, the PEGylated bilosomal suspension freezed overnight at −80 °C and was dried for a period of 24 h under vacuum [47]. The freeze-dried bilosomal powder was kept in a firmly closed glass tubes in a desiccator for further analysis.

#### 3.8.2. Differential Scanning Calorimetry (DSC)

The thermal behavior of pure **4e**, plain optimum formula and **4e**-loaded PEGylated bilosomal formula was examined adopting differential scanning calorimeter (DSC-50, Shimadzu, Kyoto, Japan). The calibration of the equipment was conducted using purified indium (99.9%). About 10 °C was elevated each minute, surrounded by nitrogen in a temperature range of 20–400 °C [48].

#### 3.8.3. Transmission Electron Microscopy (TEM)

The configuration of the optimal PEGylated bilosomal formula was visualized by TEM (Joel JEM 1230, Tokyo, Japan). The stained vesicles’ dispersion was attached to a carbon grid with copper coat and kept to dry in order to obtain a thin film. The sheet of copper was enrolled into the TEM [49].

#### 3.8.4. In Vitro Release Study of the Optimal Formula

Concisely, 1mL of sorensen phosphate buffer (pH 7.4) was gathered with 1 mL of the optimum **4e**-loaded PEGylated bilosomal formula, and then 1mL equivalent to 1 mg **4e** from the diluted dispersion was transmitted to a 10 cm in length and 2.5 cm in diameter glass cylinder. A presoaked cellulose membrane was then allocated at its bottom where the dispersion prevailed over. The glass cylinder was mounted on the shaft of the dissolution tester (Copley, DIS 8000, Nottingham, UK) and hanged in 900 mL dissolution media (sorensen phosphate buffer, pH 7.4) at 37 ± 0.5°C and speed of 50 rpm [48]. The withdrawals of equal volumes from the dissolution media were conducted at scheduled time interventions, and the percentage of drug released was assessed by HPLC at 254 nm afterwards. In vitro **4e** release was conducted in triplicates.

## 4. Conclusions

In conclusion, a series of novel acrylamide derivatives was designed and synthesized to be evaluated for their inhibitory activity against β-tubulin polymerization. The results of cytotoxicity screening over MCF-7 revealed that compounds **4e** and **5d** showed good cytotoxic profile against MCF-7 cells. Compounds **4e** produced significant reduction in cellular tubulin with excellent β-tubulin polymerization inhibition activity. In addition, compound **4e** exhibited cytotoxic activity against MCF-7 cells by cell cycle arrest at pre-G1 and G2/M phases, as shown by the DNA flow cytometry assay. Additionally, compound **4e** upregulated the expression of active caspase 3/7 percentages, as revealed by green flow cytometry analysis. PEGylated bilosomes were tailored as a nano platform for oral delivery of **4e** as a remarkable anticancer. Eight formulae were tailored in accordance to 2^3^ full factorial design. F7 was elected as the optimum formula due to the fundamentality of E.E%, PS and ZP. F7 exhibited the highest E.E%, smaller PS and absolute value of ZP. In vitro drug release studies affirmed the significant superior drug solubility of F7 over the **4e** suspension. F7 was outstanding with respect to both higher solubility and enhanced cytotoxicity. Accordingly, **4e** is developed as a lead molecule with potential anti-cancer activity, and PEGylated bilosomes can be considered as prosperous nanocarriers for compound **4e**, hence improving its biopharmaceutical characteristics and cytotoxic activity after being loaded on a vesicular nanocarrier; thus, they worthy of future investigations via in vivo preclinical investigations.

## Figures and Tables

**Table 1:** Calculated IC_50_ for compounds 2–5d against breast cancer cell line MCF-7 cells. Data expressed as the mean SE from the dose–response curve of at least three experiments.

Comp No.	IC_50_ Value (μM)	
	MCF-7	MCF-10A
**2**	>50	NT
**3**	4.73 ± 0.27	NT
**4a**	27.31 ± 1.21	NT
**4b**	10.37 ± 1.09	NT
**4c**	26.15 ± 1.21	NT
**4d**	18.37 ± 1.14	NT
**4e**	2.11 ± 0.19	29.27 ± 1.21
**5a**	3.92 ± 0.21	NT
**5b**	5.03 ± 1.01	NT
**5c**	3.03 ± 0.39	NT
**5d**	2.61 ± 0.32	NT
**Cisplatin**	1.02 ± 0.12	22.62 ± 0.19

**Table 2:** 2^3^ full factorial experimental design; experimental runs, independent variables and estimated responses of 4e-loaded PEGylated bilsomes.

Formula	A (Bile Salt Type)	B (Bile Salt Amount)	C (DSPE–mPEG-2000 Amount)	A (EE%)	B (PS)	C (ZP)	PDI
F1	SDC	30	50	85.4 ± 4.7	249.9 ± 21.5	−45.9 ± 3.8	0.25 ± 0.02
F2	STC	30	50	65.6 ± 2.3	367.6 ± 29.7	−56.7 ± 7.4	0.21 ± 0.03
F3	SDC	15	50	93.2 ± 3.6	156.5 ± 18.2	−37.8 ± 2.6	0.31 ± 0.07
F4	STC	15	50	72.6 ± 2.1	219.8 ± 13.8	−47.7 ± 2.3	0.34 ± 0.05
F5	SDC	30	25	94.4 ± 4.2	432.4 ± 21.3	−38.3 ± 3.1	0.28 ± 0.085
F6	STC	30	25	77.3 ± 3.3	527.2 ± 24.6	−50.6 ± 5.6	0.56 ± 0.08
F7	SDC	15	25	100 ± 5.6	280.3 ± 15.4	−22.5 ± 3.4	0.23 ± 0.05
F8	STC	15	25	81.5 ± 2.9	336.2 ± 18.9	−35.8 ± 3.7	0.39 ± 0.074

Notes: All values exploited as mean ± SD (*n* = 3) span 60, and cholesterol amount was constant in all formulae (100 mg and 25 mg), respectively. Abbreviations: A, entrapment efficiency percentage (EE%); B, particle size (PS); C, zeta potential (ZP); PDI, polydispersity index.

**Table 3:** 2^3^ output factorial analysis data of 4e-loaded PEGylated bilsomes and the predicted and observed responses and deviation percent of the optimum formula (F7).

Responses	EE (%)	PS (nm)	ZP (mV)
R^2^	0.9999	0.9997	0.9994
Adjusted R^2^	0.9997	0.9979	0.996
Predicted R^2^	0.9970	0.9810	0.9633
Adequate precision	172.6	73.1	53.6
Significant factors	A, B, C	A, B, C	A, B, C
Observed value of the optimal formula (F7)	100	280.3	−22.5
Predicted value of the optimal formula (F7)	99.9	282.2	−22.73
Absolute deviation %	0.1	0.68	1.02

Abbreviations: EE%, entrapment efficiency percentage; PS, particle size; ZP, zeta potential.

## Data Availability

Data is contained within the article and Supplementary material.

## Supplementary Materials

The following are available online at https://www.mdpi.com/article/10.3390/ph14101021/s1, Figure S1: The calibration curve in supplementary files.
