# Supplementary figures and images for: Design and Synthesis of Newly Synthesized Acrylamide Derivatives as Potential Chemotherapeutic Agents against MCF-7 Breast Cancer Cell Line Lodged on PEGylated Bilosomal Nano-Vesicles for Improving Cytotoxic Activity
Source: Pharmaceuticals (Basel). 2021 Oct 4;14(10):1021. doi: 10.3390/ph14101021 (PMC8540948; doi:10.3390/ph14101021)

1- The calibration curve in supplementary files

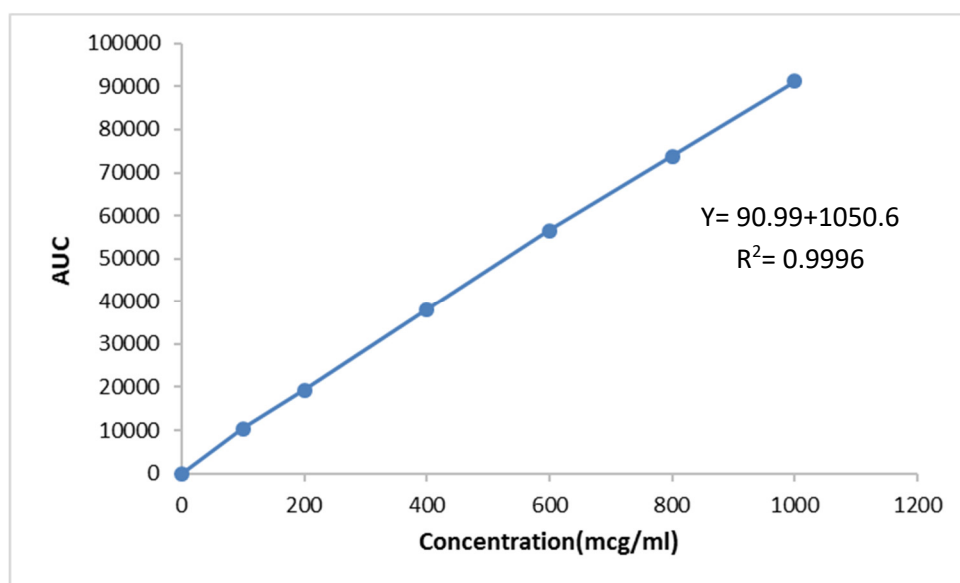

Supplement: Supplementary file 1 [file pharmaceuticals-14-01021-s001.zip › pharmaceuticals-1373743-supplementary.pdf]
